# Designing Atomically Precise and Robust COF Hybrids for Efficient Photocatalytic CO₂ Reduction

**DOI:** 10.1002/smll.202500550

**Published:** 2025-03-03

**Authors:** Laura Spies, Marcos Eduardo G. Carmo, Markus Döblinger, Zehua Xu, Tianhao Xue, Achim Hartschuh, Thomas Bein, Jenny Schneider, Antonio Otavio T. Patrocinio

**Affiliations:** ^1^ Department of Chemistry and Center for NanoScience (CeNS) University of Munich (LMU) Butenandtstraße 5–13 81377 Munich Germany; ^2^ Laboratory of Photochemistry and Materials Science (LAFOT‐CM) Institute of Chemistry Federal University of Uberlândia Uberlândia Minas Gerais 38400‐902 Brazil; ^3^ Centro de Excelência em Hidrogênio e Tecnologias Energéticas Sustentáveis – CETHS Parque Tecnológico Samambaia Goiânia Goiás 74690‐631 Brazil

**Keywords:** CO_2_ reduction, covalent organic frameworks, heterogeneous photocatalysis, hybridization

## Abstract

Hybrid photocatalysts based on molecular species and solid substrates are elegant solutions for improving the performance and stability of molecular catalytic systems aiming at solar‐driven CO_2_ conversion. In this work, a new dibenzochrysene‐based covalent organic framework (COF) is developed to accept Re^I^ centers, keeping its high crystallinity and allowing for atomistic control of the position of the catalytic centers. The rigid structure of the COF leads to long‐term stability under illumination, whereas the efficient light‐harvesting capability and the strong electronic interactions between the COF and the Re^I^ centers lead to CO evolution rates of up to 1.16 mmol g^−1^ h^−1^. The favorable photocatalytic performance of this novel Re^I^‐COF offers new insights regarding the development of efficient photocatalytic hybrid systems.

## Graphical abstract

The rational design of a novel covalent organic framework (COF)‐based hybrid CO_2_ photocatalyst allows for the atomistic level‐resolution of immobilized Rhenium(I) centers in the porous structure. The Re^I^‐decorated dibenzochrysene COF shows outstanding photocatalytic performance with a CO production rate of 1.16 mmol g^−1^ h^−1^ and long‐term photostability for 72h.

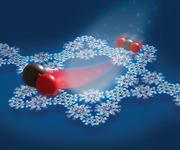

## 1 Introduction

Photocatalytic CO_2_ reduction has attracted growing interest, as it enables direct conversion of CO_2_ and solar energy into value‐added chemicals.^[^ ^1^, ^2^, ^3^ ^]^ A major breakthrough in this field was the development of the molecular rhenium(I) bipyridine (*fac*‐[Re(CO)_3_(bpy)Cl]) catalyst by Lehn and co‐workers, which opened a new avenue for designing CO_2_ photocatalysts with high precision and molecular control over reactivity.^[^ ^4^ ^]^ To leverage the tunability of molecular catalysts while addressing their inherent limitations regarding stability, hybrid photocatalytic systems have emerged, in which molecular catalysts are immobilized on solid supports such as metal oxides, nanoparticles, carbon nanostructures, and polymeric materials like g‐C_3_N_4_.^[^ ^5^, ^6^, ^7^, ^8^, ^9^ ^]^ While significant progress has been made with hybrid systems in achieving high selectivity and stability, the structural environment of the catalytic centers often remains poorly defined, with active sites randomly distributed across the substrate. This lack of atomistic control limits the potential of molecular design for optimizing the catalyst performance and hinders a comprehensive understanding of the photocatalytic CO_2_ reduction mechanism.

2D covalent organic frameworks (COFs) have been recognized as a promising platform for designing heterogeneous photocatalysts due to their virtually unlimited structural, chemical, and optoelectronic tunability.^[^ ^10^, ^11^ ^]^ COFs are crystalline porous frameworks built from organic building blocks that are connected via strong covalent bonds.^[^ ^12^ ^]^ The rational design of the building blocks and the linkage motifs results in tailor‐made materials with pre‐defined features.^[^ ^13^ ^]^ Specifically, this modular approach allows for the integration of well‐defined metal binding sites, such as bipyridine or porphyrin, which can effectively confine and stabilize CO₂ molecular catalysts, thereby facilitating rigorous structural control over hybrid photocatalysts.^[^ ^14^, ^15^, ^16^, ^17^ ^]^ Furthermore, the choice of COF linkage motifs was shown to modify the microenvironment around catalytic centers and to influence the *π*–*π* conjugation and stability of the COFs and thus their performance in photocatalytic CO_2_ reduction.^[^ ^18^, ^19^, ^20^ ^]^ Despite these advantages, synthesizing highly crystalline and stable 2D COFs is challenging due to constraints related to reaction conditions and characteristics of the building blocks.^[^ ^21^ ^]^ Achieving high crystallinity in COFs is essential not only for defining the spatial arrangement of catalytic sites but also for advancing the understanding and molecular design of hybrid photocatalysts.

**Scheme 1:**
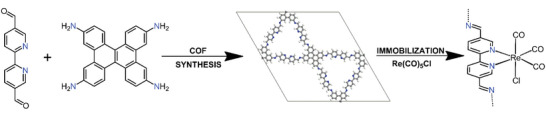
Synthesis of bpyDBC COF and immobilization of Re^I^(CO)_5_Cl, yielding Re^I^bpyDBC COF.

Here, we introduce a novel 2D COF for photocatalytic CO₂ reduction, utilizing dibenzo[g,p]chrysenetetraamine (DBC) as a structure‐directing building block for the synthesis of a highly crystalline and stable COF. DBC, a nearly planar and rigid monomer with an extended *π*‐conjugated structure, promotes tight, ordered stacking of COF layers with a high degree of conjugation. Imine‐linked DBC‐based COFs have been demonstrated to feature high crystallinity, as well as excellent thermal and chemical stability, along with long‐lived excited states.^[^ ^22^, ^23^, ^24^ ^]^ Despite these promising properties, DBC‐COFs have not yet been explored for photocatalytic applications. Here, we combine DBC with 2,2′‐bipyridine‐5,5′‐dicarbaldehyde (bpy), forming the bpyDBC COF. The bpy unit provides docking sites for the immobilization of [Re^I^(CO)_5_Cl], a model molecular CO_2_ photocatalyst (**Scheme** **1**), thereby forming the hybrid photocatalyst Re^I^bpyDBC COF. For the first time, the detailed X‐ray diffraction (XRD) and transmission electron microscopy (TEM) analysis allowed us to resolve the atomistic‐level distribution and organization of the molecular catalyst within the COF structure. The exceptional stability of the novel Re^I^bpyDBC COF under visible light illumination, maintaining performance for more than 72 h, can directly be correlated to the structural precision provided by the DBC‐node.

## 2 Results and Discussion

**Figure 1:**
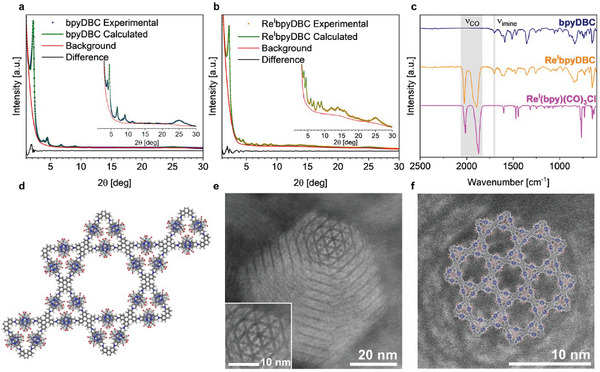
a,b) Experimental and Rietveld refined PXRD patterns of bpyDBC and Re^I^bpyDBC COFs. c) FTIR spectra of bpyDBC, Re^I^bpyDBC COFs, and the molecular analog Re^I^(bpy)(CO)_3_Cl. d) Superposition of the four structural variants of the unit cell, illustrating how the threefold propeller contrast dominated by Re^I^ is created. e) STEM‐HAADF image showing the highly ordered Re^I^ sub‐structure within the COF lattice. f) Magnified section of (e) with the COF lattice.

The synthesized bpyDBC COF was structurally analyzed using powder X‐ray diffraction (PXRD). The experimental PXRD pattern, showing sharp reflections at 2.2°, 3.9°, 4.5°, 6.8°, 8.5°, 9.0°, and 11.3° *2θ*, confirms the crystallinity of the pristine COF (**Figure** **1**a). The diffraction peak at 25° corresponds to the (001) plane, which is attributed to the *π*–*π* stacking of the as‐formed structure. For the Rietveld refinement^[^ ^25^ ^]^ of the pristine COF, we considered a combination of four fixed structural variants of the unit cell content to account for the different possible orientations of the bpy unit (Figure S2, Supporting Information). The nitrogen atoms of the bpy unit can either point into the small triangular pore (*i* and *ii*) of the Kagomé structure, or into the large hexagonal pore (*iii* and *iv*). Depending on the rotation of the imine bonds, using the DBC node as the point of reference, either a clockwise (*i* and *iii*) or anti‐clockwise (*ii* and *iv*) rotation is possible. By combining the four structural variants with equal weights of 0.25, we achieved a successful refinement of the COF in P6 symmetry and assuming AA stacking. The PXRD pattern of bpyDBC COF was fully indexed with a hexagonal lattice with refined lattice constants of a = b = 44.7(1) Å, c = 3.56(1) Å. The refined model yields a weighted R‐value (wR) of 3.49% and a profile R‐value (R_P_) of 2.48% (Table S1, Supporting Information). The highly porous nature of the COF was confirmed by nitrogen sorption measurements (Figure S3, Supporting Information) at 77 K exhibiting a Brunauer–Emmett–Teller (BET) surface area of 1252 m^2^ g^−1^ and pore sizes of 1.9 and 3.5 nm of the dual‐pore Kagomé structure.

The absence of the aldehyde and amine vibrations of the building blocks in the FTIR spectrum, along with the appearance of the imine stretching vibration at 1690 cm^−1^, confirm the formation of the covalent bond (Figure S4, Supporting Information). Additional evidence is provided by solid‐state ^13^C CP‐MAS‐NMR (Figure S5, Supporting Information).

The Re^I^‐centers were introduced into the framework following a well‐established procedure based on heating of the COF and the [Re(CO)_5_Cl] precursor under under reflux in an inert atmosphere.^[^ ^16^, ^20^ ^]^ Upon the introduction of Re^I^, the crystallinity of the COF network is preserved and additional diffraction peaks appear at 5.9°, 8.0°, 9.9°, 12.0°, and 13.9°, attributed to an increase of the corresponding structure factors (Figure 1b). For the Rietveld refinement, the model containing the respective Re^I^‐loaded structural variants *i_Re –_ iv_Re_* (Figure S2, Supporting Information) yields wR = 2.66% and a profile R‐value (R_P_) of 2.08% (Table S1, Supporting Information), with a refined occupation factor of the bpy sites with Re^I^(CO)_3_Cl of 47%. This is close to the value of 44% (16.2 wt.% Re) obtained by ICP OES. Similar refined lattice constants are obtained with a = b = 44.8(1) Å, c = 3.65(1) Å. Nitrogen sorption experiments of the Re^I^‐loaded COF revealed a BET surface area of 717 m^2^ g^−1^ and pore sizes of 1.9  and 3.3 nm (Figure S3, Supporting Information). The successful immobilization of [Re^I^(CO)_3_Cl] by the bpyDBC COF was further evidenced by the three CO vibrational bands observed between 1800 and 2100 cm^−1^ in the FTIR, corresponding to the A_1_ and the two E modes expected for the *facial* geometry of the Re^I^(CO)_3_Cl moiety,^[^ ^26^, ^27^ ^]^ and resembling those of the molecular catalyst [Re^I^(bpy)(CO)_3_Cl] (Figure 1c). Thermogravimetric analysis (TGA) of bpyDBC COF and Re^I^bpyDBC COF shows good thermal stability, with decomposition temperatures of up to 420 and 300 °C, respectively (Figure S6, Supporting Information). SEM images were taken to examine the morphology of the pristine and modified COFs (Figure S7, Supporting Information). The bpyDBC COF shows rod‐like structures growing from spherical particles that cluster into larger domains, while the Re^I^‐functionalized COF has similar spherical particles covered with platelet‐like structures.

To investigate the distribution of the Re^I^‐complex within the COF structure at the near‐atomic level, TEM analysis was performed. TEM images of bpyDBC COF and Re^I^bpyDBC COF (Figures S8a, S9a, Supporting Information, respectively) are similar, showing a fully crystallized, homogenous material with crystallite sizes between 50 and 100 nm. Several research groups have shown that the bpy units in COF networks can serve as binding sites for metal complexes, however, the spatial resolution of the distribution of metal centers within the COF framework has yet to be demonstrated.^[^ ^16^, ^17^, ^20^, ^28^ ^]^ Employing scanning TEM in high‐angle annular dark field mode (STEM‐HAADF), we were able to resolve, for the first time, the position of the metal centers in the crystalline COF network. Here, the image contrast is approximately proportional to the atomic number squared, making heavy atoms such as Re^I^ appear as bright spots. STEM‐HAADF images of the Re^I^bpyDBC COF reveal a highly ordered Re^I^ sub‐structure (Figure 1e; Figure S9b–d, Supporting Information), adopting the crystallographic positions defined by the bpy linker. The superposition of the four structural variants (*i*_Re_)–(*iv*_Re_) (Figure 1d) used for the Rietveld refinement illustrates how a statistical distribution of the Re^I^ centers in the COF lattice creates the threefold propeller‐type pattern visible in the STEM‐HAADF image (Figure 1f). The high crystallinity and well‐defined structural properties of the bpyDBC COF enabled the direct observation of the arrangement of the metal centers within the COF framework at a near‐atomic scale. This unprecedented level of resolution underscores the pivotal role of structural order in facilitating the precise localization of metal centers, which is essential for understanding their catalytic behavior and optimizing photocatalytic performance.

**Figure 2:**
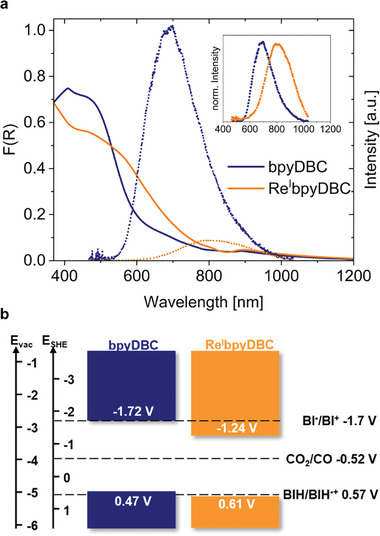
a) UV–vis absorption (continuous line, smoothed) and PL emission (dotted line, λ_exc_ = 476 nm) of bpyDBC and Re^I^bpyDBC COFs. The normalized PL intensity of the two COFs is shown in the inset. b) Energy levels of the two COFs determined by CV and Tauc plot analysis, and reduction potentials of the sacrificial donor BIH and the CO_2_‐to‐CO reduction.

It is well established that the incorporation of metal centers affects the electronic properties of organic frameworks.^[^ ^29^, ^30^ ^]^ Hence, changes in the electronic structure of the COF upon immobilization of the Re^I^ complex were investigated via UV–vis and photoluminescence (PL) measurements along with cyclic voltammetry (CV). The optical absorption onset of Re^I^bpyDBC COF is redshifted by ≈150 nm compared to bpyDBC COF. Fitting this onset with a Tauc plot (Figure S10, Supporting Information) yields direct bandgaps of 2.19 eV for bpyDBC COF and 1.85 eV for Re^I^bpyDBC COF. Similar redshifts upon the incorporation of the Re^I^‐complex have been reported in the literature.^[^ ^16^, ^17^, ^20^ ^]^ By calculating the band structure of metal‐doped bpy‐based COFs, Kamiya *et al.* found that the 3d orbitals of the metal are located below the conduction band of the COF, thus causing the optical redshift.^[^ ^30^ ^]^ The PL emission of the pristine COF is characterized by a broad band with λ_*em*_ = 693 nm, which is significantly quenched in the Re^I^bpyDBC COF, indicating the efficient electronic interaction between the COF and the Re^I^ centers (**Figure** **2**a). Time‐resolved measurements with time‐correlated single‐photon counting (TCSPC) reveal a multi‐component decay similar to those observed by Pan and coworkers for a different Re‐COF hybrid (Figure S11, Supporting Information).^[^ ^14^ ^]^ Double exponential fits of the experimental signals reveal lifetimes of 32 (77%) and 241 ps (23%) for the pristine bpyDBC COF and 30 (82%) and 227 ps (18%) for Re^I^bpyDBC COF. The precision of such values, especially the shortest lifetimes, is affected by the response function of the TCSPC setup, but the data suggest the increase of non‐radiative processes in the Re^I^bpyDBC hybrid, likely due to the charge transfer from the COF to the Re^I^ centers.

To estimate the valence band positions of the pristine and modified COFs, CV was performed in accordance with previously reported studies.^[^ ^31^, ^32^, ^33^ ^]^ By fitting the onset of the first reversible oxidation peak (Figure S12, Supporting Information), valence band energies of bpyDBC COF and Re^I^bpyDBC COF were determined to be 0.47 and 0.61 V versus SHE, respectively. Combining CV and UV–vis results, the conduction band minimum energies of the two materials were estimated (Figure 2b), establishing that both COFs are thermodynamically capable of reducing CO_2_ to CO. In addition to thermodynamic suitability for catalytic conversion, the incorporation of Re^I^ centers into the COF significantly enhanced CO_2_ uptake, thereby facilitating the interactions between the hybrid catalyst and the reactant (Figure S13, Supporting Information).

In the photocatalytic CO_2_ reduction assays, 1,3‐dimethyl‐2‐phenyl‐2,3‐dihydro‐1H‐benzimidazole (BIH) was used as a sacrificial electron donor. BIH has a stronger reduction potential (*E_ox_* = 0.57 V vs SHE)^[^ ^34^ ^]^ compared to other commonly used sacrificial electron donors such as TEA (triethylamine, *E_ox_* = 0.693 V vs SHE)^[^ ^35^ ^]^ TEOA (triethanolamine, *E_ox_* = 1.06 V vs SHE)^[^ ^36^ ^]^ or BNAH (1‐benzyl‐1,4‐dihydronicotinamide, *E_ox_* = 0.81 V vs SHE)^[^ ^37^ ^]^ and, moreover, upon oxidation, BIH undergoes rapid deprotonation to form the radical BI^•^, which is an even stronger reducing agent (*E_p_* = −1.7 V vs SHE)^[^ ^34^ ^]^ (Figure 2b). This allows BIH to donate two electrons and one proton during the photocatalytic process, as shown in Scheme S3 (Supporting Information), thereby boosting the CO_2_ conversion. Additionally, the fully oxidized form of BI^+^ does not negatively impact the photocatalytic CO_2_ reduction.^[^ ^38^ ^]^ The performance of Re^I^bpyDBC COF in the photocatalytic CO_2_ reduction was investigated using a 300 W Xe lamp as the illumination source equipped with 370 or 400 nm long‐pass filters. In both cases, the irradiance was set at 100 mW cm^−2^. Acetonitrile suspensions containing the COF catalyst and a 24‐fold molar excess of BIH were exposed to illumination under a saturated CO_2_ atmosphere. The gaseous products were monitored by gas chromatography.

**Figure 3:**
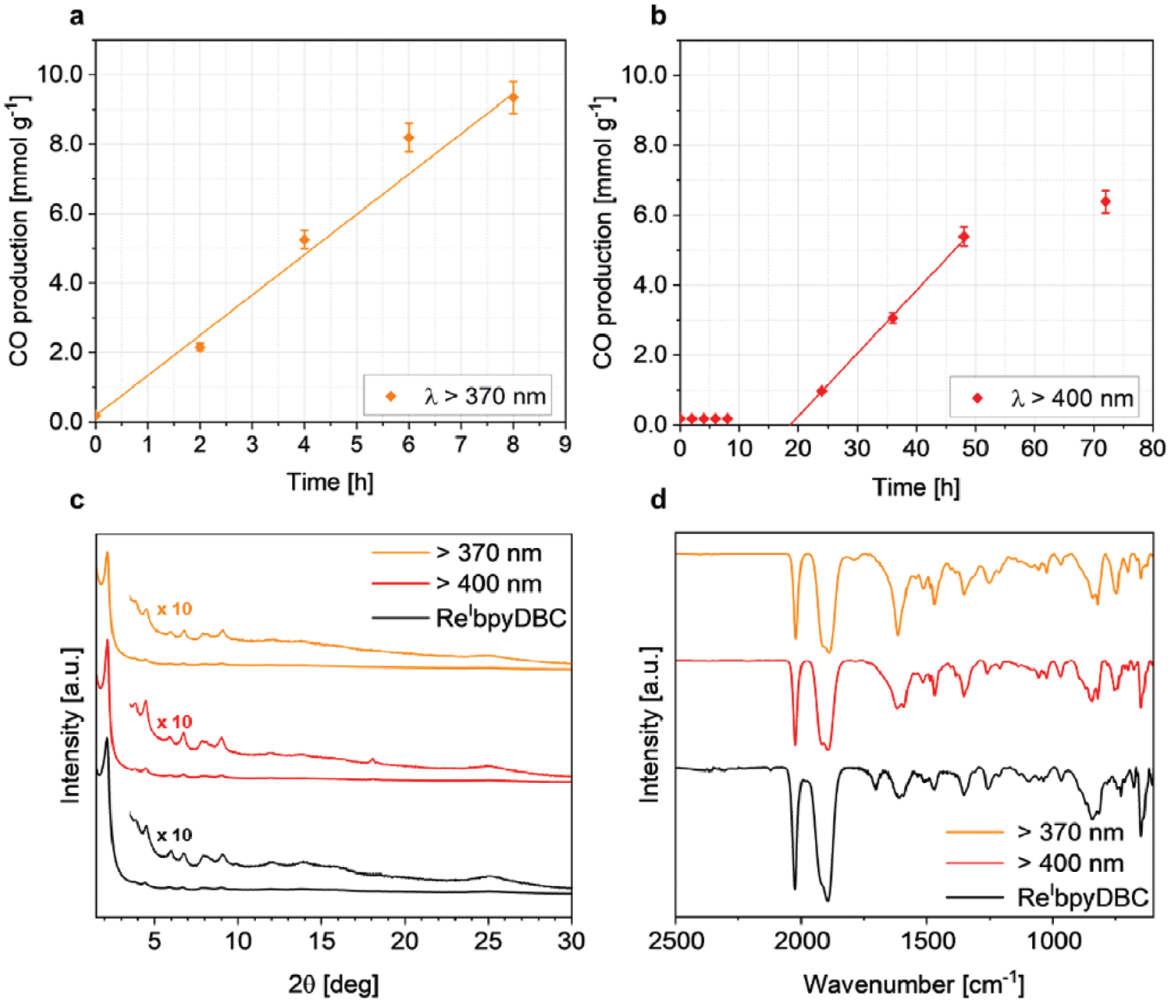
CO production of the Re^I^bpyBC COF under illumination with a) λ > 370 nm and b) λ > 400 nm. Solid lines represent the linear fitting of the data to extract CO production rates. PXRD pattern c) and FTIR spectra d) of the Re^I^bpyDBC COF before and after 24 h of photocatalysis under both illumination conditions.

Notably, in the photocatalytic experiments, CO was identified as the only reduction product. Control experiments conducted under argon atmosphere, in the dark, and in the absence of a catalyst did not yield any CO. Experiments using isotopically labeled ^13^CO_2_ yielded ^13^CO for both illumination conditions (Figures S14, S15, Supporting Information), evincing that the CO originated from the reduction of CO_2_. **Figure** **3**a displays the CO evolution using Re^I^bpyDBC COF as a photocatalyst under UV–vis (>370 nm) illumination. A remarkably high rate of 1.16 mmol g^−1^ h^−1^ was achieved, which ranks amongst the highest CO rates reported in the literature for similar systems without the use of an additional photosensitizer.^[^ ^16^, ^17^, ^20^ ^]^ A comparison of the reported rates can be found in Table S2 (Supporting Information), however, comparing rates from different groups is non‐intuitive and should be approached with caution, as differences in the photocatalytic setup (e.g., wavelength of source, photon flux, reactor geometry, catalyst concentration, stirring, etc.) can significantly influence the catalyst performance.^[^ ^39^, ^40^ ^]^ In contrast, the pristine bpyDBC COF did not show any CO evolution under these conditions, underlining the importance of the Re^I^ centers serving as the active sites in the COF structure. Recycling studies carried out under UV–vis light reveal that after 8 h illumination, a new purging with CO_2_ leads to the production of more CO (Figure S16, Supporting Information), leading to a maximum turnover number (TON_CO_) of 35. PXRD and FTIR measurements conducted after the reaction confirm the structural integrity of the Re^I^bpyDBC COF following 24 h of UV–vis illumination (Figure 3c,d, orange line).

Under visible light (> 400 nm) illumination, an evolution rate of 0.18 mmol g^−1^ h^−1^ was achieved after an induction period of ≈ 20 h  (Figure 3b). The different behavior under different illumination conditions indicates distinct mechanisms of CO_2_ conversion. UV–vis light can directly induce MLCT excitation of the molecular catalyst followed by reductive quenching through BIH and the release of the Cl^−^‐ligand. This yields the well‐known penta‐coordinated 18‐electron species driving the CO_2_ to CO conversion.^[^ ^41^ ^]^ In contrast, under visible light excitation, light harvesting primarily involves internal *π*–*π* ^*^ transitions within the highly conjugated bpyDBC COF structure. Subsequent electron transfer from the COF to the catalytic Re^I^ centers is required to generate the catalytically active 18‐electron species. The efficiency of this electron transfer is most likely limited by the intrinsic charge carrier dynamics of the COF. Consistent with previously reported in‐situ spectroscopic studies for Re‐COFs, the ≈20 h induction period observed here under visible‐light excitation is consequently attributed to the stepwise accumulation of the catalytically active species, which initiates CO_2_ conversion once a critical concentration is achieved.^[^ ^16^ ^]^

We do not attribute the onset of CO production after the induction period to any structural changes or degradation of the COF material. Post‐reaction PXRD and FTIR measurements (Figure 3c,d, red line) collected after 24 h of illumination (i.e., after the onset of CO production) show no evidence of changes in the chemical structure of the COF or the coordination environment of the Re^I^ centers. Additionally, the ¹^3^CO labeling experiments validate that the CO product originates from CO₂ reduction rather than decomposition of the material.

The outstanding stability of the Re^I^bpyDBC COF is further evidenced by its continuous CO production for 72 h under visible light. In contrast, a homogeneous catalyst, released through degradation of the hybrid COF‐catalyst, would be unlikely to sustain such prolonged activity.

Unlike previously reported COFs, where the loss of crystallinity led to ceased photoactivity,^[^ ^20^ ^]^ the Re^I^bpyDBC COF exhibits exceptional stability, maintaining its catalytic performance for 72 h – approximately three times longer than the longest‐reported Re‐COF system under standard photocatalytic conditions (Table S3, Supporting Information). This outstanding stability can be directly correlated to the extremely high crystallinity, the precise definition of the local coordination environment as well as excellent electronic delocalization achieved through the use of the structure‐directing DBC‐node.

## 3 Conclusion

Concluding, in this study we demonstrate that the favorable low curvature and large conjugated structure of the dibenzochrysene‐based building unit are key factors for enhancing both the crystallinity and stability of the novel hybrid photocatalyst Re^I^bpyDBC COF for CO_2_ reduction. The bpyDBC COF provides predefined binding sites for the immobilization of Re^I^(CO)_5_Cl, yielding structural motifs of the model catalyst *fac*‐Re(CO)_3_(bpy)Cl throughout the framework. For the first time, we successfully resolved the highly ordered sub‐structure adopted by the Re^I^ centers within the COF using STEM‐HAADF analysis, providing valuable insights regarding the molecular design of hybrid catalysts. In the photocatalytic CO_2_‐to‐CO conversion, the Re^I^bpyDBC COF exhibited a remarkable CO production rate of 1.16 mmol g^−1^ h^−1^ under UV–vis illumination with BIH serving as a sacrificial electron donor. Under visible light, the Re^I^‐COF maintained catalytic activity for over 72 h at a reduced rate of 0.18 mmol g^−1^ h^−1^, competing with the longest operational lifetime of other COF‐based CO_2_ photocatalysts. Our work highlights that COFs can function as effective platforms for the hybridization of molecular catalysts, offering atomistic control over structure and exceptional stability.

## Conflict of Interest

The authors declare no conflict of interest.

## Supporting information



Supporting Information

## Data Availability

The data that support the findings of this study are available in the supplementary material of this article.

## References

[1] S. Fang , M. Rahaman , J. Bharti , E. Reisner , M. Robert , G. A. Ozin , Y. H. Hu , Nat. Rev. Methods Primers 2023, 3, 61.

[2] J. Albero , Y. Peng , H. García , ACS Catal. 2020, 10, 5734.

[3] E. Karamian , S. Sharifnia , J. CO2 Util. 2016, 16, 194.

[4] J. Hawecker , J.‐M. Lehn , R. Ziessel , J. Chem. Soc., Chem. Commun. 1983, 536.

[5] X. Qiao , Q. Li , R. N. Schaugaard , B. W. Noffke , Y. Liu , D. Li , L. Liu , K. Raghavachari , L.‐S. Li , J. Am. Chem. Soc. 2017, 139, 3934. 28271885 10.1021/jacs.6b12530

[6] R. Kuriki , K. Sekizawa , O. Ishitani , K. Maeda , Angew. Chem. Int. Ed. Eng. 2015, 54, 2406. 10.1002/anie.201411170 25565575

[7] N. M. Muresan , J. Willkomm , D. Mersch , Y. Vaynzof , E. Reisner , Angew. Chem. 2012, 124, 12921. 10.1002/anie.201207448 23169697

[8] S. E. Maier , T. Nagel , M. Turan , E. Kaya , W. Frey , M. Dyballa , D. P. Estes , Organometallics 2024, 43, 233.

[9] G. N. Silva , L. A. Faustino , L. L. Nascimento , O. F. Lopes , A. O. T. Patrocinio , J. Chem. Phys. 2024, 160, 034701. 38226823 10.1063/5.0178945

[10] M. E. G. Carmo , L. Spies , G. N. Silva , O. F. Lopes , T. Bein , J. Schneider , A. O. T. Patrocinio , J. Mater. Chem. A 2023, 11, 13815.

[11] J. You , Y. Zhao , L. Wang , W. Bao , J. Clean. Prod. 2021, 291, 125822.

[12] M. S. Lohse , T. Bein , Adv. Funct. Mater. 2018, 28, 1870229.

[13] N. Keller , T. Bein , Chem. Soc. Rev. 2021, 50, 1813. 33331358 10.1039/d0cs00793e

[14] Q. Pan , M. Abdellah , Y. Cao , W. Lin , Y. Liu , J. Meng , Q. Zhou , Q. Zhao , X. Yan , Z. Li , H. Cui , H. Cao , W. Fang , D. A. Tanner , M. Abdel‐Hafiez , Y. Zhou , T. Pullerits , S. E. Canton , H. Xu , K. Zheng , Nat. Commun. 2022, 13, 845. 35149679 10.1038/s41467-022-28409-2 PMC8837612

[15] M. Lu , J. Liu , Q. Li , M. Zhang , M. Liu , J. L. Wang , D. Q. Yuan , Y. Q. Lan , Angew. Chem., Int. Ed. 2019, 58, 12392. 10.1002/anie.201906890 31270914

[16] S. Yang , W. Hu , X. Zhang , P. He , B. Pattengale , C. Liu , M. Cendejas , I. Hermans , X. Zhang , J. Zhang , J. Huang , J. Am. Chem. Soc. 2018, 140, 14614. 30352504 10.1021/jacs.8b09705

[17] S.‐Y. Li , S. Meng , X. Zou , M. El‐Roz , I. Telegeev , O. Thili , T. X. Liu , G. Zhu , Microporous Mesoporous Mater. 2019, 285, 195.

[18] S. Suleman , K. Sun , Y. Zhao , X. Guan , Z. Lin , Z. Meng , H.‐L. Jiang , CCS Chem. 2024, 6, 1689.

[19] Y. Xiang , W. Dong , P. Wang , S. Wang , X. Ding , F. Ichihara , Z. Wang , Y. Wada , S. Jin , Y. Weng , H. Chen , J. Ye , Appl. Catal., B 2020, 274, 119096.

[20] Z. Fu , X. Wang , A. M. Gardner , X. Wang , S. Y. Chong , G. Neri , A. J. Cowan , L. Liu , X. Li , A. Vogel , R. Clowes , M. Bilton , L. Chen , R. S. Sprick , A. I. Cooper , Chem. Sci. 2020, 11, 543. 32206271 10.1039/c9sc03800k PMC7069507

[21] F. Haase , B. V. Lotsch , Chem. Soc. Rev. 2020, 49, 8469. 33155009 10.1039/d0cs01027h

[22] N. Keller , T. Sick , N. N. Bach , A. Koszalkowski , J. M. Rotter , D. D. Medina , T. Bein , Nanoscale 2019, 11, 23338. 31793601 10.1039/c9nr08007d

[23] Z. Xie , B. Wang , Z. Yang , X. Yang , X. Yu , G. Xing , Y. Zhang , L. Chen , Angew. Chem. Int. Ed. Eng. 2019, 58, 15742. 10.1002/anie.201909554 31433550

[24] T. Xue , R. Guntermann , A. Biewald , D. Blätte , D. D. Medina , A. Hartschuh , T. Bein , ACS Appl. Mater. Interfaces 2024, 16, 48085. 39193985 10.1021/acsami.4c09286

[25] B. H. Toby , R. B. Dreele , J. Appl. Crystallogr. 2013, 46, 544.

[26] M. K. Itokazu , A. S. Polo , D. L. A. Faria , C. A. Bignozzi , N. Y. M. Iha , Inorg. Chim. Acta 2001, 313, 149.

[27] L. A. Faustino , B. L. Souza , B. N. Nunes , A.‐T. Duong , F. Sieland , D. W. Bahnemann , A. O. T. Patrocinio , ACS Sustain. Chem. Eng. 2018, 6, 6073.

[28] W. Zhong , R. Sa , L. Li , Y. He , L. Li , J. Bi , Z. Zhuang , Y. Yu , Z. Zou , J. Am. Chem. Soc. 2019, 141, 7615. 30998334 10.1021/jacs.9b02997

[29] J. Chen , X. Tao , L. Tao , H. Li , C. Li , X. Wang , C. Li , R. Li , Q. Yang , Appl. Catal., B 2019, 241, 461.

[30] T. Hosokawa , M. Tsuji , K. Tsuchida , K. Iwase , T. Harada , S. Nakanishi , K. Kamiya , J. Mater. Chem. A 2021, 9, 11073.

[31] J. M. Rotter , R. Guntermann , M. Auth , A. Mähringer , A. Sperlich , V. Dyakonov , D. D. Medina , T. Bein , Chem. Sci. 2020, 11, 12843. 34094480 10.1039/d0sc03909h PMC8163307

[32] R. Guntermann , L. Frey , A. Biewald , A. Hartschuh , T. Clark , T. Bein , D. D. Medina , J. Am. Chem. Soc. 2024, 146, 15869. 38830115 10.1021/jacs.4c02365

[33] R. Guntermann , D. Helminger , L. Frey , P. M. Zehetmaier , C. Wangnick , A. Singh , T. Xue , D. D. Medina , T. Bein , Angew. Chem., Int. Ed. 2024, 63, 202407166. 10.1002/anie.202407166 39138128

[34] Y. Tamaki , K. Koike , T. Morimoto , O. Ishitani , J. Catal. 2013, 304, 22.

[35] Y. L. Chow , W. C. Danen , S. F. Nelsen , D. H. Rosenblatt , Chem. Rev. 1978, 78, 243.

[36] A.‐M. Manke , K. Geisel , A. Fetzer , P. Kurz , Phys. Chem. Chem. Phys. 2014, 16, 12029. 24556846 10.1039/c3cp55023k

[37] S. Fukuzumi , S. Koumitsu , K. Hironaka , T. Tanaka , J. Am. Chem. Soc. 1987, 109, 305.

[38] Y. Kuramochi , O. Ishitani , H. Ishida , Coord. Chem. Rev. 2018, 373, 333.

[39] M. Schwarze , D. Stellmach , M. Schröder , K. Kailasam , R. Reske , A. Thomas , R. Schomäcker , Phys. Chem. Chem. Phys. 2013, 15, 3466. 23361354 10.1039/c3cp50168j

[40] G. V. Akhil , M. S. Ramyashree , A. Veekshit Udayakumar , S. S. Priya , K. Sudhakar , T. Muhammad , J. Ind. Eng. Chem. 2021, 99, 19.

[41] H. Takeda , K. Koike , H. Inoue , O. Ishitani , J. Am. Chem. Soc. 2008, 130, 2023. 18205359 10.1021/ja077752e

