# Supplementary material for: Designing Atomically Precise and Robust COF Hybrids for Efficient Photocatalytic CO₂ Reduction
Source: Small. 2025 Mar 3;21(26):2500550. doi: 10.1002/smll.202500550 (PMC12232230; doi:10.1002/smll.202500550)
Supplement: Supplementary file 1 — Supporting Information [file SMLL-21-2500550-s001.docx]

**Supporting Information**

**Designing Atomically Precise and Robust COF Hybrids for Efficient Photocatalytic CO2 Reduction**

Laura Spies^a‡^, Marcos Eduardo G. Carmo^b‡^, Markus Döblinger^a^, Zehua Xu^a^, Tianhao Xue^a^, Achim Hartschuh^a^, Thomas Bein^a*^, Jenny Schneider^a*^ and Antonio Otavio T. Patrocinio^b,c*^

^a^Department of Chemistry and Center for NanoScience (CeNS), University of Munich (LMU), Butenandtstraße 5-13, 81377 Munich, Germany

^b^Laboratory of Photochemistry and Materials Science, Institute of Chemistry, Federal University of Uberlândia, Uberlândia, MG, Brazil

^c^Centro de Excelência em Hidrogênio e Tecnologias Energéticas Sustentáveis – CETHS, Parque Tecnológico Samambaia, 74690-631, Goiânia, GO, Brazil

**Table of Content**

[S1. Materials and Methods 2](#_Toc187925649)

[S2. Synthetic Procedures 5](#_Toc187925650)

[S3. Rietveld Refinement 8](#_Toc187925651)

[S4. Structural and Electronic Characterization of bpyDBC and Re^I^bpyDBC COF 10](#_Toc187925652)

[Nitrogen Sorption experiments 10](#_Toc187925653)

[FTIR Spectroscopy 10](#_Toc187925654)

[MAS NMR 11](#_Toc187925655)

[Thermogravimetric Analysis 12](#_Toc187925656)

[Electron Microscopy 13](#_Toc187925657)

[Tauc Plot of Absorption Onset 15](#_Toc187925658)

[Cyclic Voltammetry 15](#_Toc187925659)

[CO_2_ Sorption Experiment 16](#_Toc187925660)

[S5. Photocatalytic experiments 17](#_Toc187925661)

[Reaction scheme of sacrificial donor 17](#_Toc187925662)

[Comparison of photocatalytic performance 17](#_Toc187925663)

[^13^CO_2_ isotope labelling experiments 19](#_Toc187925664)

[Recycling studies 21](#_Toc187925665)

# S1. Materials and Methods

All solvents were purchased from *Sigma Aldrich*, *Acros* or *TCI Europe* in the common purities *purum*, *puriss* or *reagent grade*. The materials were used as received without additional purification and handled in air unless otherwise noted.

**Powder X-ray diffraction** (PXRD) measurements were performed using a Bruker D8 Discover instrument with Ni-filtered Cu K_α_ radiation and a LynxEye position-sensitive detector.

**Fourier-transform infrared spectroscopy** (FTIR) measurements were performed with a Bruker Vertex 70 FTIR instrument by focusing the light of a globar as a MIR light source through a KBr beam splitter with integrated gold mirrors and an ATR sample stage with a Ge crystal. The spectra were recorded with an N_2_ cooled MCT detector at a resolution of 2 cm^−1^ and averaged over 128 scans.

**Scanning electron microscopy** (SEM) micrographs were obtained with an FEI Helios NanoLab G3 UC microscope equipped with a Schottky field-emission electron source operated at 3 kV.

**Transmission electron microscopy** (TEM) and scanning TEM in high-angle annular dark-field mode (STEM-HAADF) was performed on a probe-corrected FEI Titan Themis instrument equipped with a field emission gun operated at 300 kV.

**Nitrogen sorption isotherms** were recorded with a Quantachrome Autosorb 1 instrument at 77 K. **Carbon dioxide sorption** measurements were conducted on a Quantachrome Autosorb iQ at 273 K and 298 K. The samples were outgassed for 24 h at 120°C under high vacuum prior to the measurements. Pore size distributions based on the nitrogen sorption data were calculated using a QSDFT model with a carbon kernel for cylindrical pores.

**Nuclear magnetic resonance** (NMR) spectra were recorded on Bruker AV 400 and AV 400 TR
spectrometers. Proton chemical shifts are expressed in parts per million (*δ* scale) and are
calibrated using residual non-deuterated solvent peaks as internal reference (e.g., DMSO-*d*_6_: 2.50 ppm).

**UV-Vis-NIR** spectra were recorded on a Perkin-Elmer Lambda 1050 spectrometer equipped with a 150 mm integrating sphere, photomultiplier tube (PMT) and InGaAs detector. **Diffuse reflectance spectra** were collected with a Praying Mantis (Harrick) accessory and were referenced to barium sulfate powder as 100%R standard. The specular reflection of the sample surface was removed from the signal using apertures that allow only light scatter at angles > 20° to pass. Diffuse reflectance data was smoothed using the LOWESS filter, integrated in the Origin 2022b data analysis software.

**Steady-state photoluminescence** (PL) and **time-correlated single-photon counting** (TCSPC).
A home-built confocal laser scanning microscope (CLSM) setup was used for characterizing the photoluminescence of the COF samples. The samples were measured in the epi-direction using an air objective (0.85 NA, Fluor 40, NIKON). A beamsplitter (MELLES GRIOT 03BTL005) and a 490 nm long-pass filter were utilized to separate the laser from the photoluminescence (PL) light. Excitation was provided by a sub-picosecond laser (iChrome TOPTICA) operating at 476 nm with a repetition rate of 40 MHz. The detection system was divided into two components. The first part featured an avalanche photodiode (APD, type: MPD PDM, with a detector size of 50 × 50 μm), which was used in combination with time-correlated single-photon counting (TCSPC) electronics (BECKER UND HICKEL) to measure time-resolved PL transients. The second part comprised a spectrometer (ANDOR SHAMROCK SRi303) connected to a CCD camera (ANDOR NEWTON DU920) for capturing spectra. The data were recorded using a customized LABVIEW (National Instruments) program that integrated the manufacturers’ software with our specific measurement requirements. Further data processing and analysis, including extracting PL spectra and TCSPC transients, were performed using MATLAB (MATHWORKS).

**Thermogravimetric analysis** (TGA) measurements were performed using a Netzsch Jupiter ST 449 C instrument equipped with a Netzsch TASC 414/4 controller. The samples were heated from room temperature to 900°C under a synthetic air flow (25 mL min^-1^) at a heating rate of 10 K min^-1^.

**Cyclic Voltammetry** was performed on a Metrohm Autolab Potentiostat using a three-electrode setup with Pt wire as counter electrode, Ag wire as pseudo-reference electrode and slurry-coated FTO glass as working electrode in 0.1M TBAPF_6_ in acetonitrile. The electrolyte was prepared and stored under Ar. The Ag pseudo-reference electrode was referenced against the ferrocene redox couple. Measured potentials *vs.* the ferrocene redox couple Fc/Fc^+^ were converted to the absolute electrode potential (*E_vac_*) and to potentials *vs*. Standard Hydrogen Electrode (SHE) according to the following equations (i) and (ii), respectively. Note that the absolute potential scale equals to the negative physical scale.^[1]^ Potentials *vs*. Standard Calomel Electrode (SCE) were converted to potentials *vs.* SHE according to equation (iii).^[2]^ Reduction potentials for the sacrificial donors retrieved from the literature were converted to SHE accordingly.

1. $E_{vac}= E_{{Fc/Fc}^{+}}+4.8\mathrm{eV}$
2. $E_{vac}=E_{SHE}+4.44\mathrm{eV}$
3. $E_{SHE}=E_{SCE}+0.241V$

Slurries for electrode coating were prepared by dispersing 21 mg of COF material, 4.5 mg Ketjen black and 4.5 mg PVDF (70:15:15) in 300 µL NMP. The mixture was repeatedly vortexed and ultrasonicated until the desired viscosity was achieved. 15 µL of the slurry was then drop-cast onto FTO glass and dried immediately using a heat gun.

**Photocatalytic experiments** were carried out in a quartz cuvette with a total volume of 5.08 mL. The reactor was loaded with 3.50 mL of acetonitrile, 1.0 mg of photocatalyst and 5.0 mg of BIH. The mixture was then purged with CO_2_ until saturation was reached and exposed to a 300 W Xe lamp equipped with a water filter and a 370 or 400 nm long-pass filter. At given time intervals, 100 μL of the headspace atmosphere was sampled to quantify the amount of produced CO using a gastight syringe and analyzed using **Gas Chromatography** (GC) (Shimadzu GC2014), equipped with a flame ionization detector (FID) and a methanizer. A PerkinElmer Clarus 580 – GC-TCD was employed to check potential H_2_ production.
**Stability experiments** were conducted under the same conditions as the photocatalytic experiments. After 24 h, the COF powder was removed from the reaction suspension *via* filtration, washed with small amounts of THF and subsequently dried under vacuum.

**Gas Chromatography-Mass Spectrometry** (GC-MS). To reveal the carbon source in the photoreaction product, namely carbon monoxide (CO), ^13^C isotope labelled CO_2_ gas (Sigma Aldrich, 99 atom % ^13^C, < 3 atom % ^18^O) was used in the photocatalytic reaction following the same procedure described above. In this case, a gas sample (500 μL) from the headspace of the cuvette was analysed by GC-MS Shimadzu QP2020-NX with electron ionisation (EI) equipped with a U-Bond column (30 m, ID 0.32 mm) for ^13^CO_2_ separation and a Molsieve 5A column (30 m, ID 0.32mm) for ^13^CO separation without N_2_ interference. The standard gas mixture of CO_2_, CO, CH_4_, O_2_, and N_2_ as balance (All-in-gas GmbH) was used to set the instrument method and to identify the analytes’ retention times (RT). The characteristic ions at RT_CO_ = 6.7 min with m/z 29 (^13^C^16^O), 28 (^12^C^16^O), 16 (^16^O), and 13 (^13^C) were detected in single ion monitoring mode (SIM). Shimadzu Postrun Data Analysis software was used to process the data.

# S2. Synthetic Procedures

**Synthesis of COF linker molecules.** The COF linker 2,2’-bipyridine-5,5’-dicarbaldehyde (bpy) was purchased from BLDpharm. Dibenzo[g,p]chrysenetetraamine, (DBCTA) was synthesized according to the literature.^[3]^ The ^1^H NMR spectrum of the compound is shown in Figure S1.


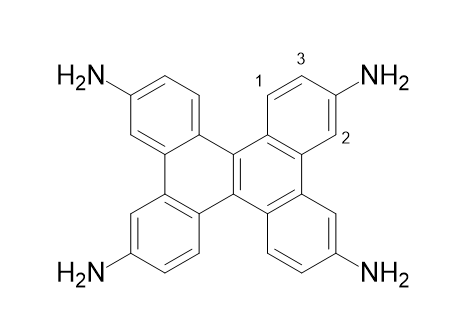

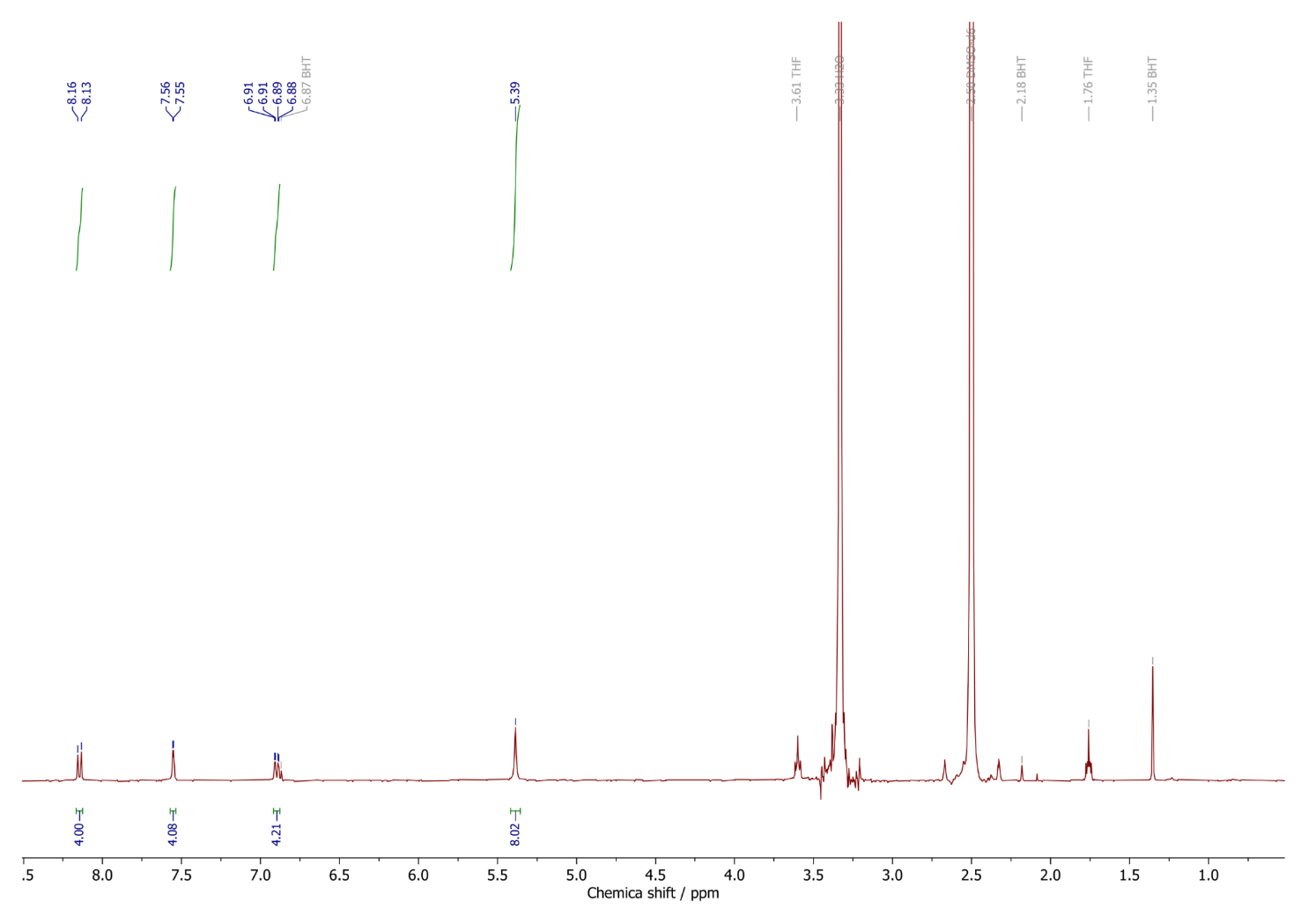


Figure S1. ^1^H NMR spectrum of DBCTA: ^1^H NMR (400 MHz, DMSO-*d*_6_) *δ* (ppm): 8.15 (d, *J* = 8.8 Hz, 4H, C(1)H), 7.55 (d, *J* = 2.3 Hz, 4H, C(2)H), 6.9 (dd, *J* = 8.8, 2.3 Hz, 4H, C(3)H), 5.39 (s, 8H, NH_2_).


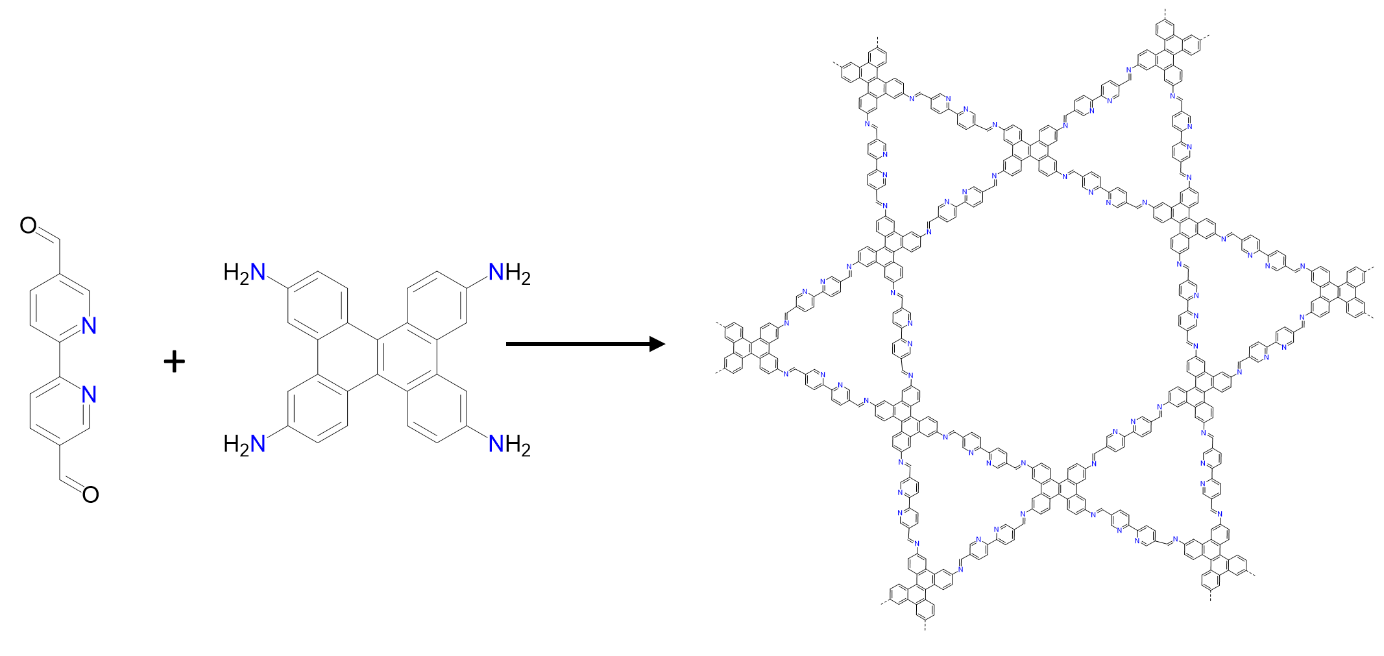


Scheme S1. Scheme of the synthesis route for bpyDBC COF.

**Synthesis of bpyDBC COF.** In a 6 mL Schott-Duran culture tube, a solid mixture of 2,2’-bipyridine-5,5’-dicarbaldehyde, bpy(CHO)_2_ (6.38 mg, 0.03 mmol) and dibenzo[g,p]chrysenetetraamine, DBCTA (5.84 mg, 0.015 mmol) was suspended in 500 µL 1,4-dioxane and mesitylene (250 µL each). Subsequently, 50 µL of acetic acid (aqueous, 6 M) was added. The culture tube was tightly sealed and heated at 120°C for 4 days. The resulting suspension was filtered and the isolated brown powder was Soxhlet-extracted with THF for 8 h, followed by extraction in supercritical CO_2_ at 110 bar and 40°C for 1 hour. Elemental analysis for (C_50_H_28_N_8_)*_n_*, calculated: C, 81.07; H, 3.81; N, 15.13. Found: C, 69.94; H, 4.27; N, 12.62. Calculations for the elemental analysis were based on an infinite structure, ignoring the presence of undefined end-groups.

**Synthesis of Re^I^bpyDBC COF.** BpyDBC COF (30 mg) and [Re(CO)_5_Cl] (14.65 mg, 0.04 mmol) were dispersed in 15 mL toluene and heated to reflux for 40 min. The resulting dark brown powder was filtered hot and washed thoroughly with methanol. The resulting powder was dried under vacuum. ICP-OES analysis was conducted to determine the Re content to 15.87 ± 0.43 wt%, which equals an occupation of bpy sites of 43.5 ± 2.7 %. Despite meticulous execution of experiments, the range of Re content varies slightly for each batch. The batch used for Rietveld analysis and STEM-HAADF contained 16.2 wt% Re with a bpy occupation of 43.9 %, the batch for the photocatalytic measurements contained 16.91 wt% Re, which equals to 0.93 mmol Re g^-1^ and an occupation of the bpy sites of 47 %. The percentage of bpy sites occupied by Re^I^(CO)₃Cl is calculated based on the following considerations:

To estimate the molecular weight of the COF, a fragment reflecting the 2:1 ratio of the bpy to the DBC linker is considered (Scheme S2). The molecular weight of this C_50_H_28_N_8_^4+^ fragment is M = 740.83 g mol^-1^.


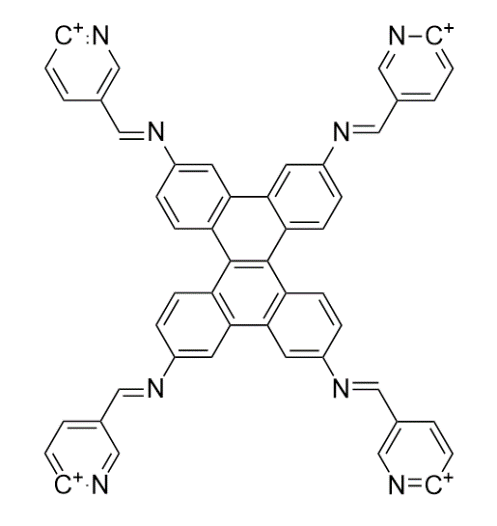
The weight percent of rhenium given by the ICP-OES can be expressed as the following equation:

$$y=\frac{x*M(\mathrm{Re})}{M\left( C_{50}H_{28}N_{8}^{4+} \right)+x*M(\mathrm{Re}\left( \mathrm{CO} \right)_{3}Cl)}$$

where $x$ is a number between 0 and 2, corresponding to the number of immobilized Re-complexes per fragment (max. value = 2, since only two bpy sites are available per fragment). A 100% coverage of the two available bpy sites of the fragment translates to two additional Re^I^(CO)_3_Cl compounds, each with a weight of M = 305.69 g mol^-1^, thus 2 in the equation. Insertion of the corresponding molecular weights (M(Re) = 182.21 g mol^-1^) yields a value of 0.269, or 26.9 wt% for a 100% coverage of the bpy sites with Re-complex.

Scheme S2. COF fragment reflecting the 2:1 ratio of bpy to DBC.

In reality, the value obtained by ICP-OES is used to calculate the coverage of bpy sites. For example, ICP-OES measures 16.2 wt% Re in the sample of interest. Replacing 0.162 in the above equation and solving it for yields a value of 0.878. Applying the rule of three, if 2 corresponds to 100%, then 0.878 corresponds to coverage of 43.9% of the bpy sites.

**Synthesis of sacrificial donor.** The benzimidazolidine derivate, BIH (1,3-dimethyl-2-phenylbenzo[d]imidazolidine) was synthesized as previously reported.^[4]^ ^1^H NMR (CDCl_3_ 400 MHz) δ (ppm): 7.61–7.53 (m, 2H), 7.45–7.37 (m, 3H), 6.71 (dd, J = 5.4, 3.2 Hz, 2H), 6.43 (dd, J = 5.4, 3.2 Hz, 2H), 4.88 (s, 1H), 2.56 (s, 6H).

# S3. Rietveld Refinement

Molecular models were constructed by connecting the bpy and DBC building blocks, resulting in the hexagonal Kagomé connectivity shown in Scheme 1. As indicated by STEM-HAADF imaging, the Re atoms can be both oriented towards the small pore or the large pore of the COF. The bpy unit can face either into the small trigonal or into the large hexagonal pore of the Kagomé structure and the imine bonds can be rotated either clockwise or anti-clockwise with respect to the DBC node. We combined the four resulting variations *(i)* – *(iv)* for bpyDBC and *(i_Re_)* - *(iv_Re_)* for Re^I^bpyDBC, reflecting extreme cases (Figure S12), thereby expecting to exhaustively cover the space of possible distributions.

Powder X-ray diffraction patterns of bpyDBC and Re^I^bpyDBC were fully indexed with hexagonal lattices and refined lattice constants of a = b = 44.7(1) Å, c = 3.74(1)Å (bpyDBC) and a = b = 44.8(1) Å, c = 3.65(1)Å (Re^I^bpyDBC). For the Rietveld refinement^[5]^ of bpyDBC the four variants (Figure S2) were used with a fixed weight of 0.25, fixed atomic positions and constrained to have equal lattice parameters, crystallite sizes and Re^I^ fractions.

Table S1. Structural parameters of the two COFs and reliability factors of the Rietveld refinement.

|  | **bpyDBC** | **Re^I^bpyDBC** |
| --- | --- | --- |
| **Space group** | P6 | P6 |
| **a, b** | 44.7(1) | 44.8(1) |
| **c** | 3.561(4) | 3.65(1) |
| **Re^I^** | ---- | 0.468(4) |
| **wR** | 3.49 % | 2.66 % |
| **Rp** | 2.48 % | 2.08 % |


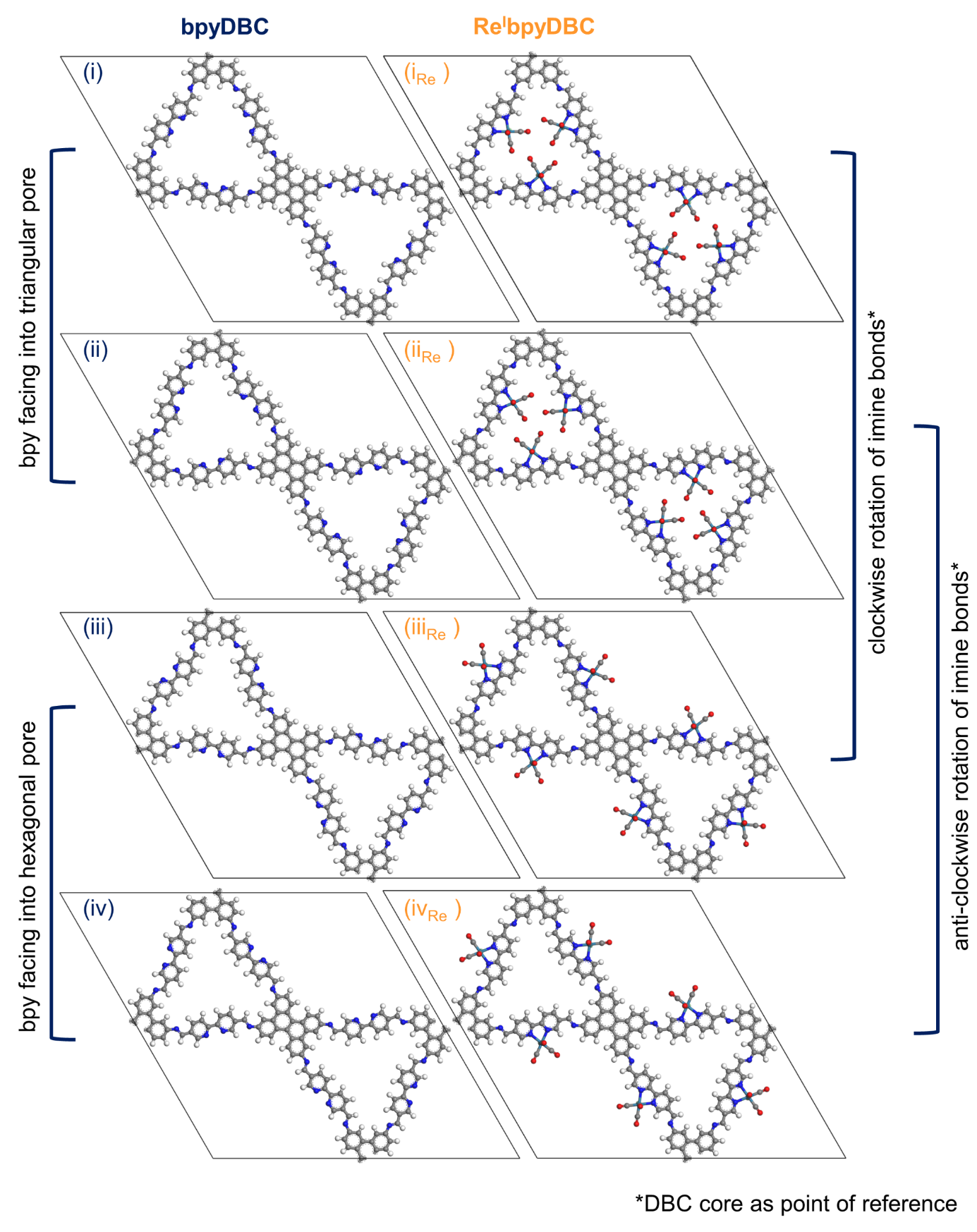


Figure S2. Structural variants of the two COFs.

# S4. Structural and Electronic Characterization of bpyDBC and Re^I^bpyDBC COF

## Nitrogen Sorption experiments


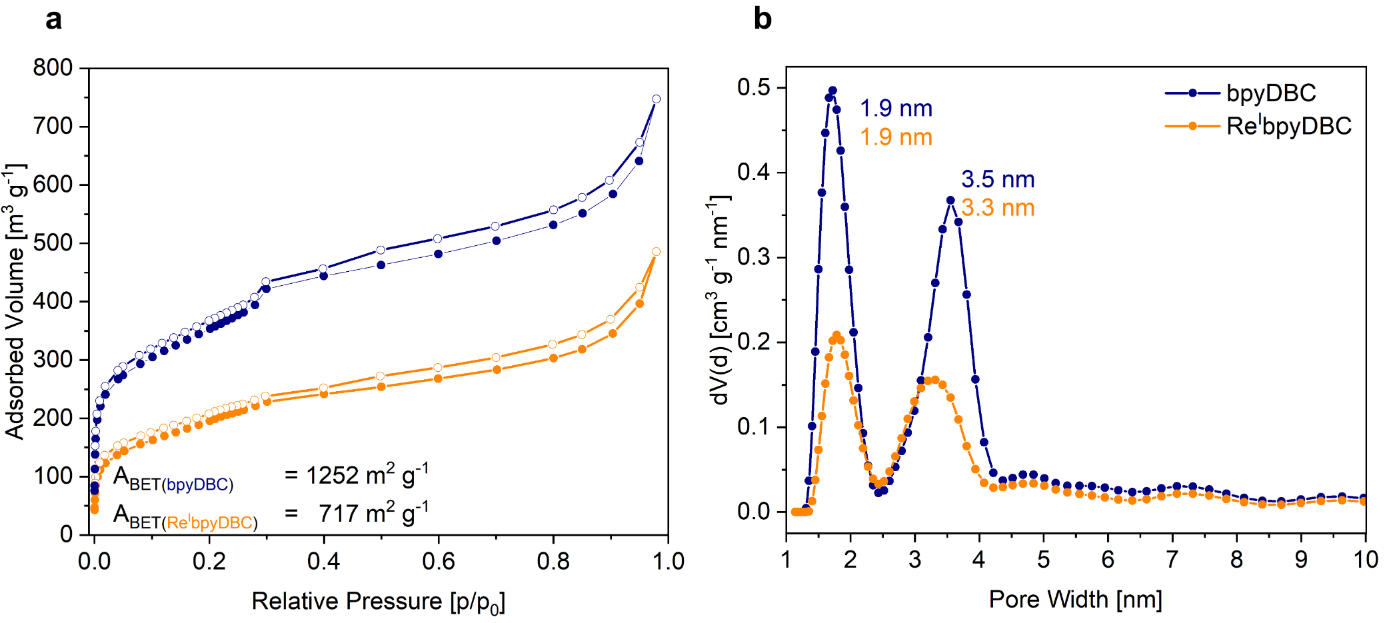


Figure S3. (a) Nitrogen sorption isotherms of bpyDBC and Re^I^bpyDBC COF and (b) pore size distributions of both COFs.

## FTIR Spectroscopy


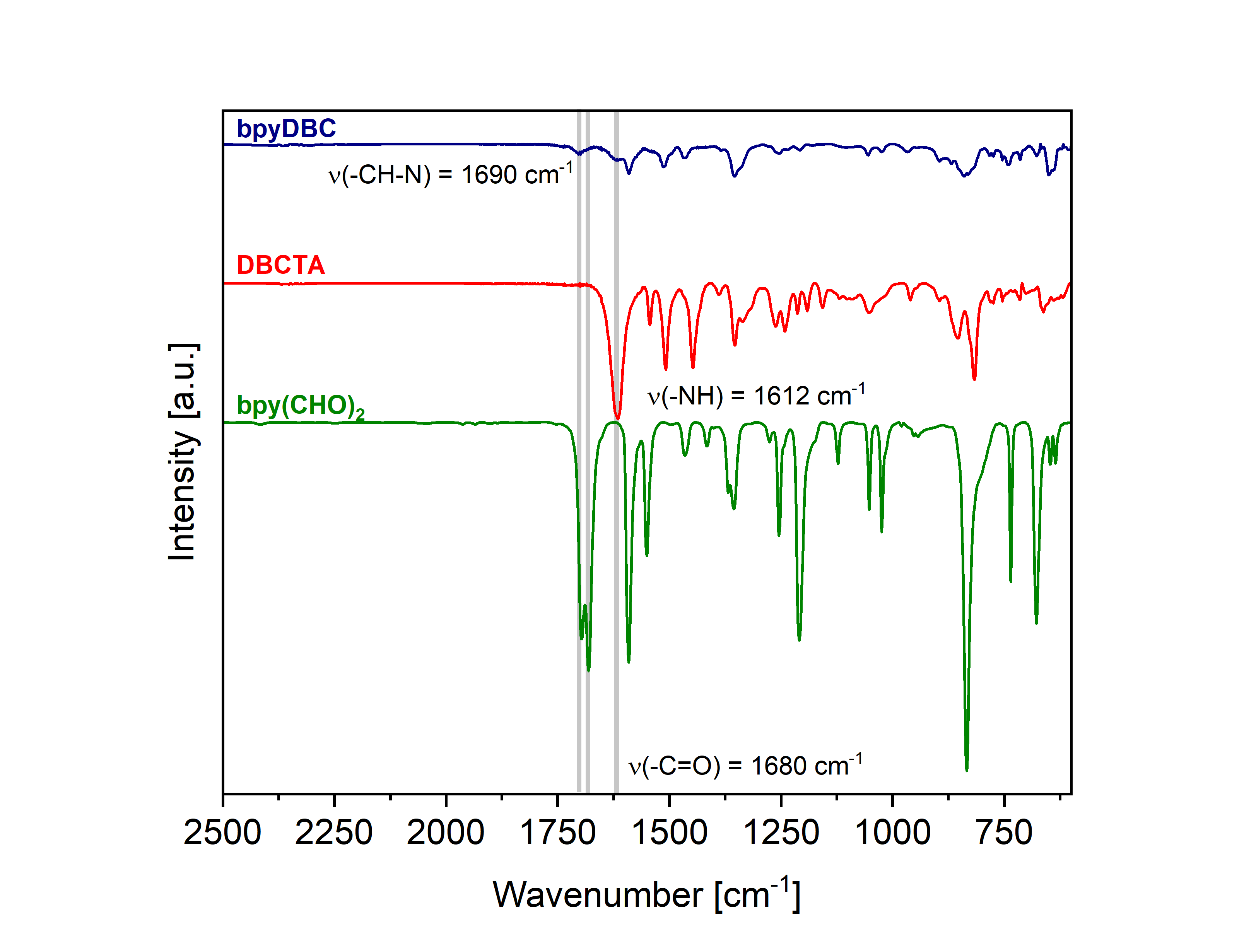


Figure S4. FTIR spectra of the two linkers bpy(CHO)_2_ and DBCTA together with the spectrum of the bpyDBC COF.

## MAS NMR


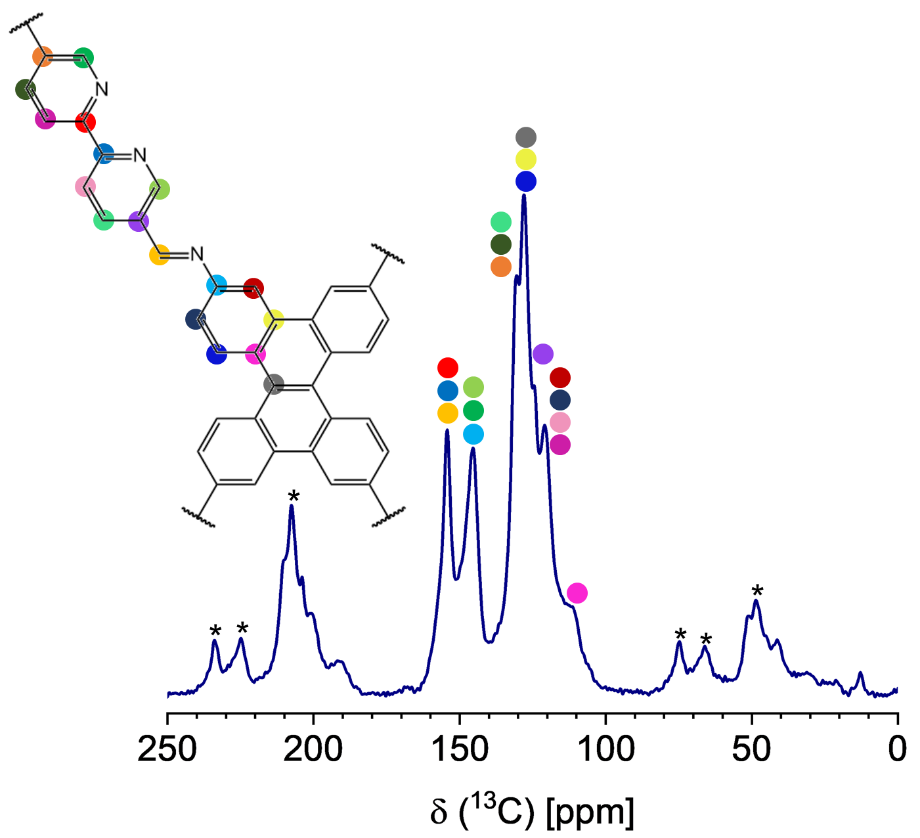


Figure S5: Solid-state ^13^C CP-MAS-NMR spectrum of bpyDBC COF. The absence of a peak corresponding to the aldehyde functions at approximately 190 ppm as well as the presence of a peak at approximately 155 ppm corresponding to the imine bond confirms the formation of the imine-linked framework. The peaks were assigned to the most probable carbon atoms. The signals marked with an asterisk correspond to spinning sidebands.

## Thermogravimetric Analysis


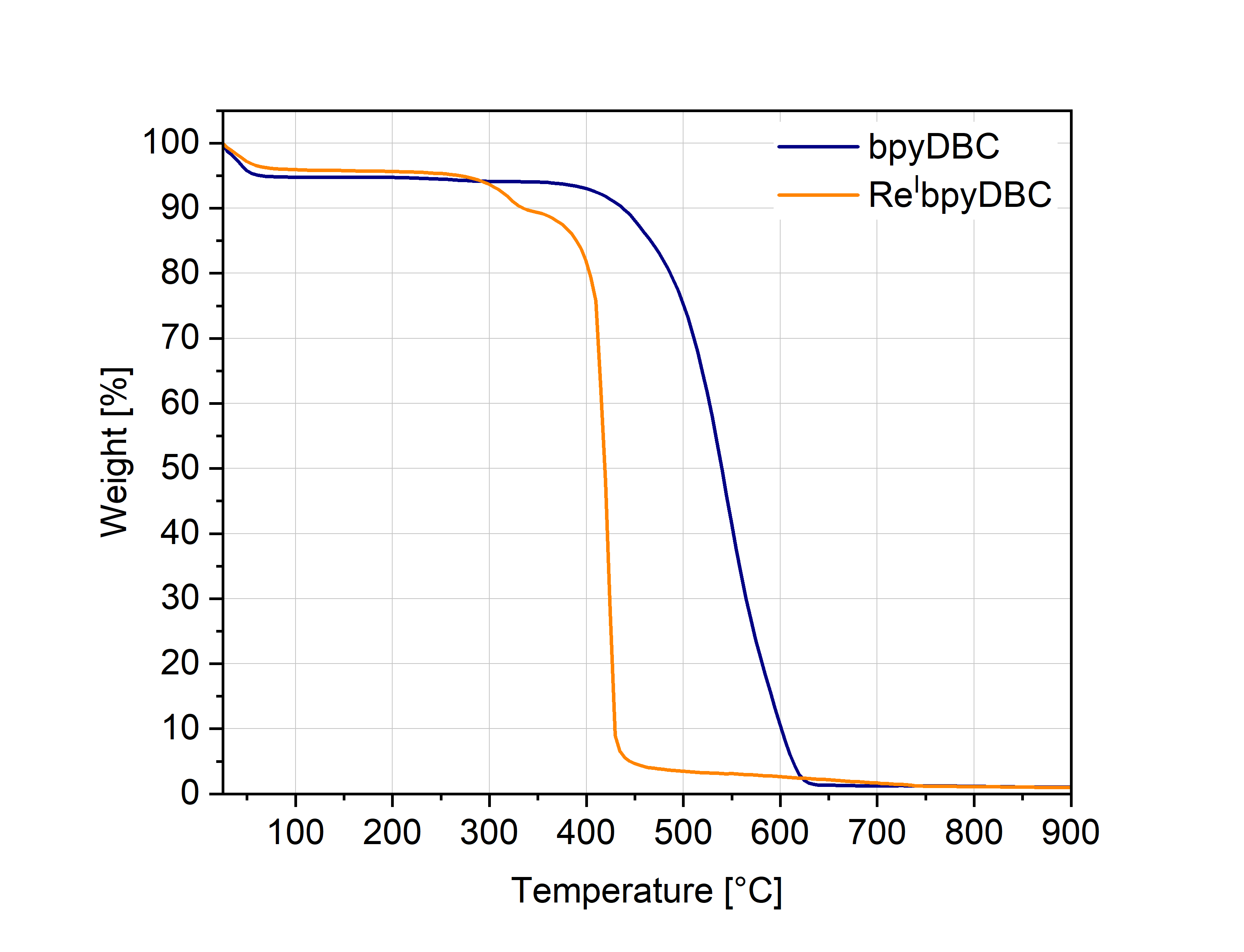


Figure S6. TGA of bpyDBC and Re^I^bpyDBC, measured under a synthetic air flow (25 mL min^-1^) at a heating rate of 10 K min^-1^.

## Electron Microscopy


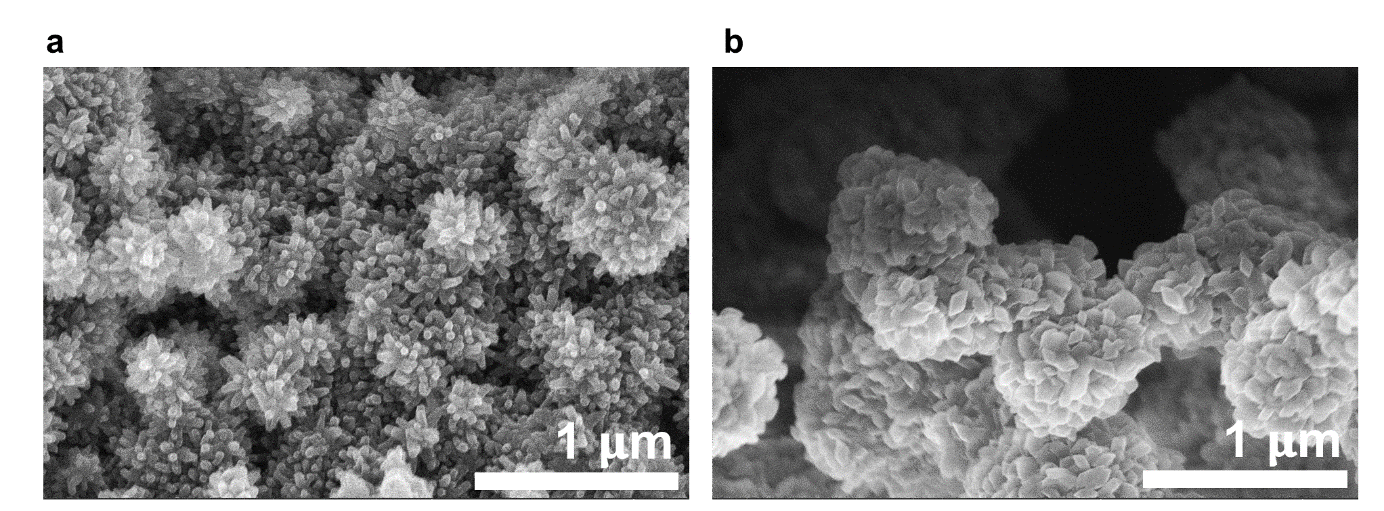


Figure S7. SEM micrographs of (a) bpyDBC and (b) Re^I^bpyDBC COF.

TEM images confirm the crystallinity of the COFs, with domain sizes of 50-100 nm for both bpyDBC and Re^I^bpyDBC (Figures S8a and S9a, respectively). STEM-HAADF images of the pristine COF are not expected to provide further insights since the light atoms of the COF are only weakly scattering into large angles and the COF is further prone to beam damage (Figure S8b). In contrast, the highly ordered, periodic Re^I^ substructure of the Re^I^bpyDBC COF is clearly observed by STEM-HAADF throughout the sample. Figure S9b-d shows example STEM-HAADF images of the Re^I^bpyDBC COF.


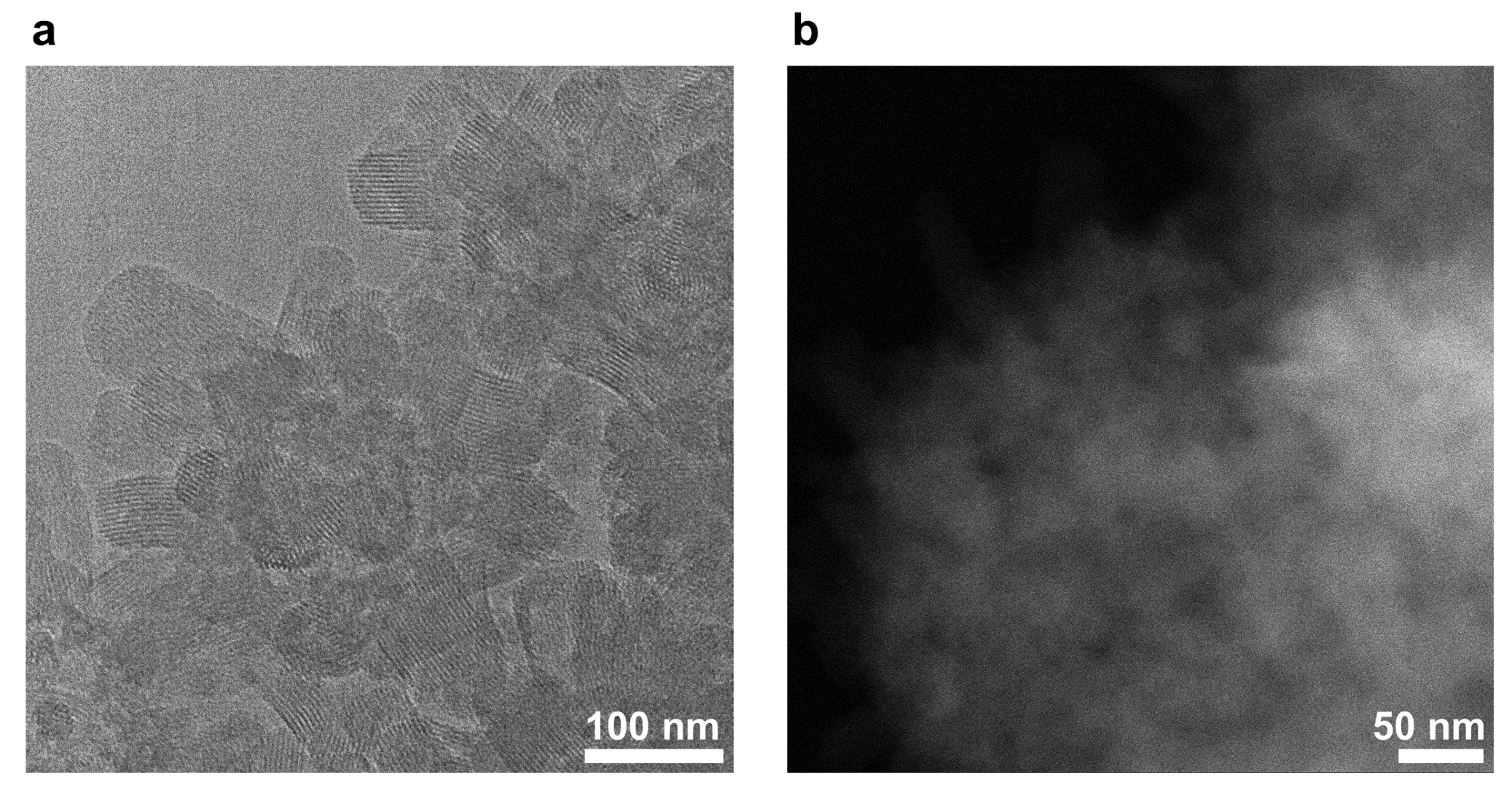


Figure S8. (a) TEM and (b) STEM-HAADF images of the bpyDBC COF.


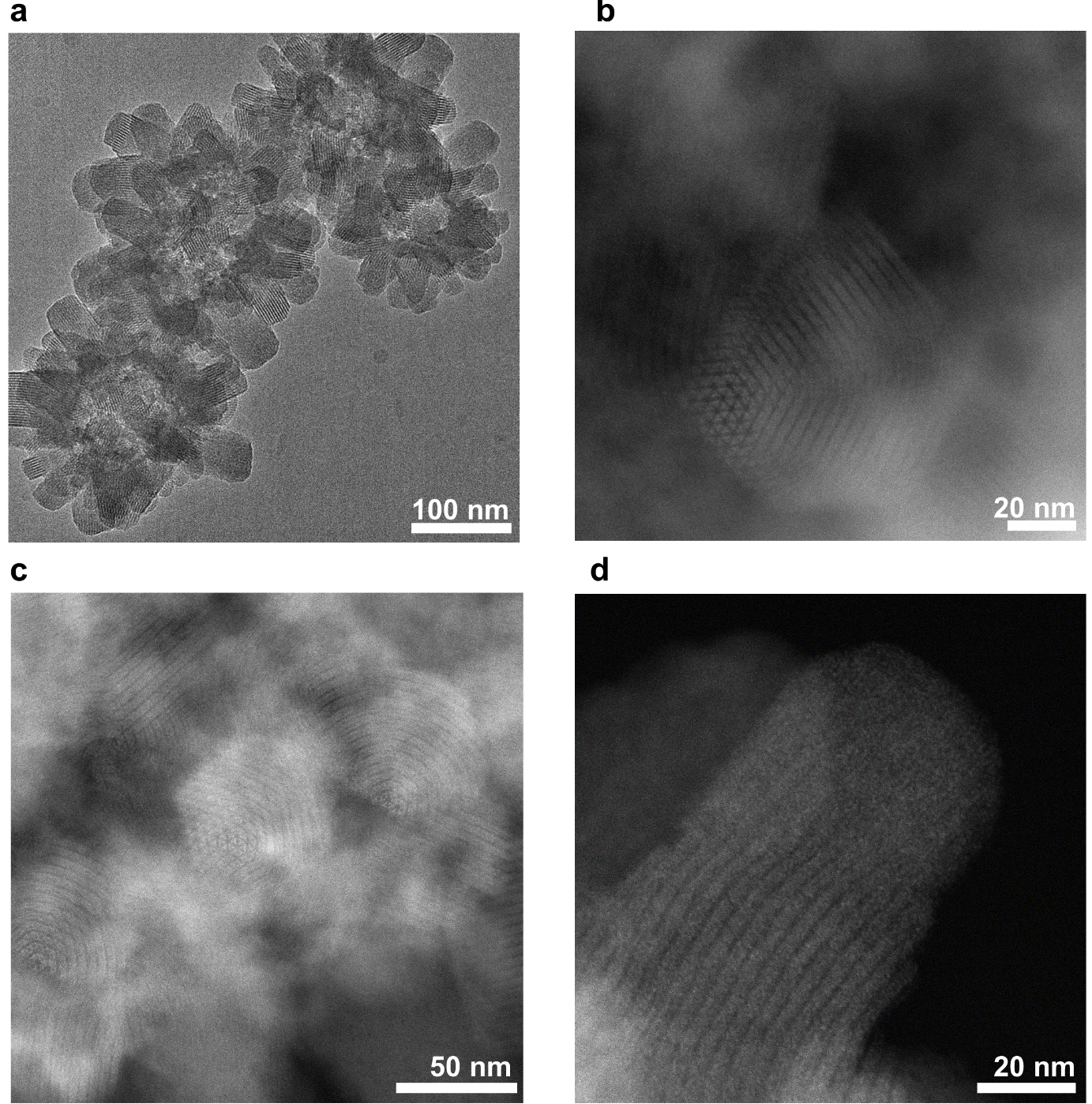


Figure S9. (a) TEM image and (b) – (d) STEM-HAADF images of the Re^I^bpyDBC COF taken at different positions and magnifications.

## Tauc Plot of Absorption Onset


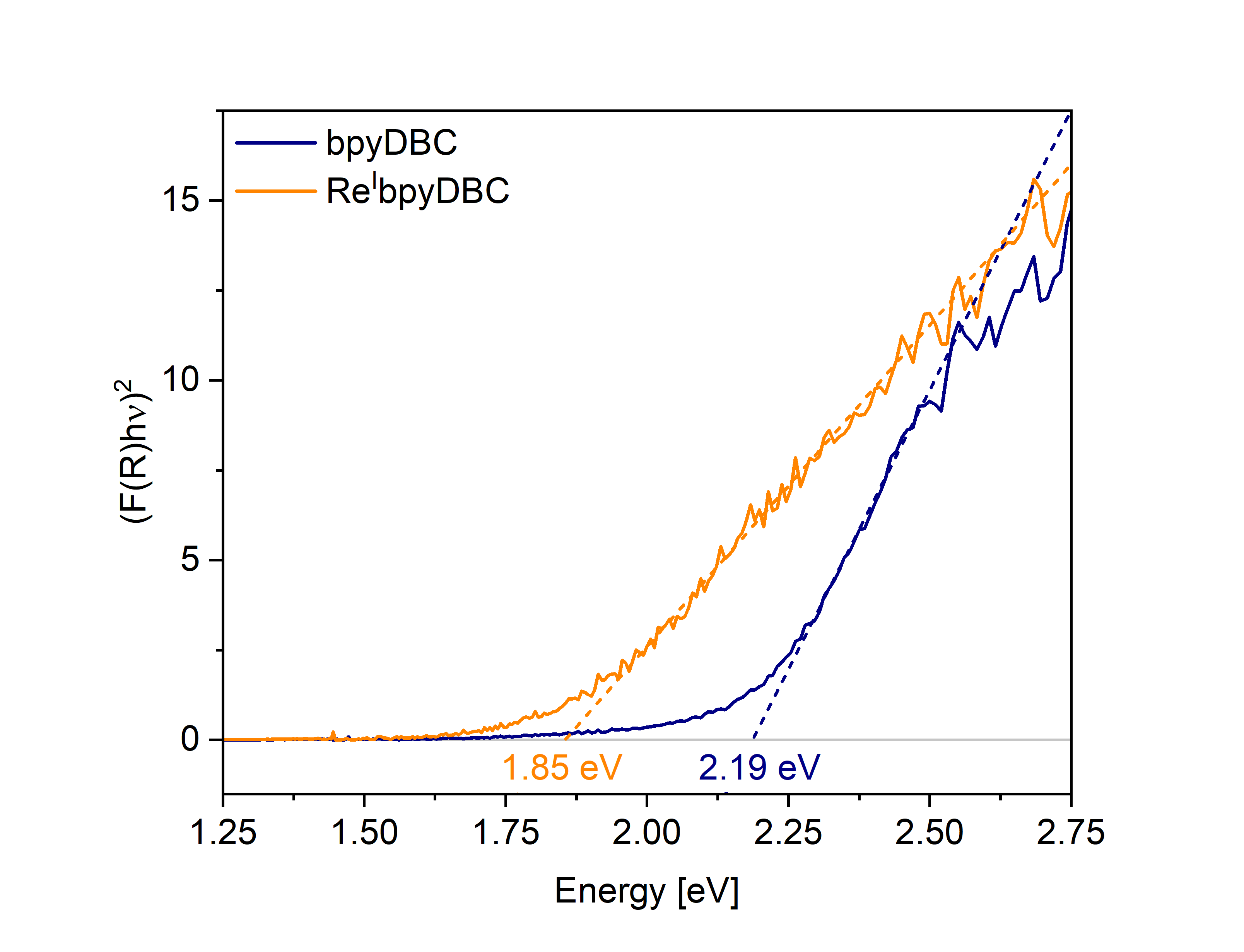


Figure S10. Tauc plot of the two COFs assuming a direct transition. The band gap energy is reduced upon introduction of the Re-complex into the COF structure.

Figure S11. Excited state lifetime of the bpyDBC and Re^I^bpyDBC COF measured using the TCSPC method (λ_exc_ = 476 nm) and collected at the maximum emission wavelength. The COFs were measured as dry powders under ambient conditions. The signals were fitted to a double-exponential decay (red lines).

## Cyclic Voltammetry


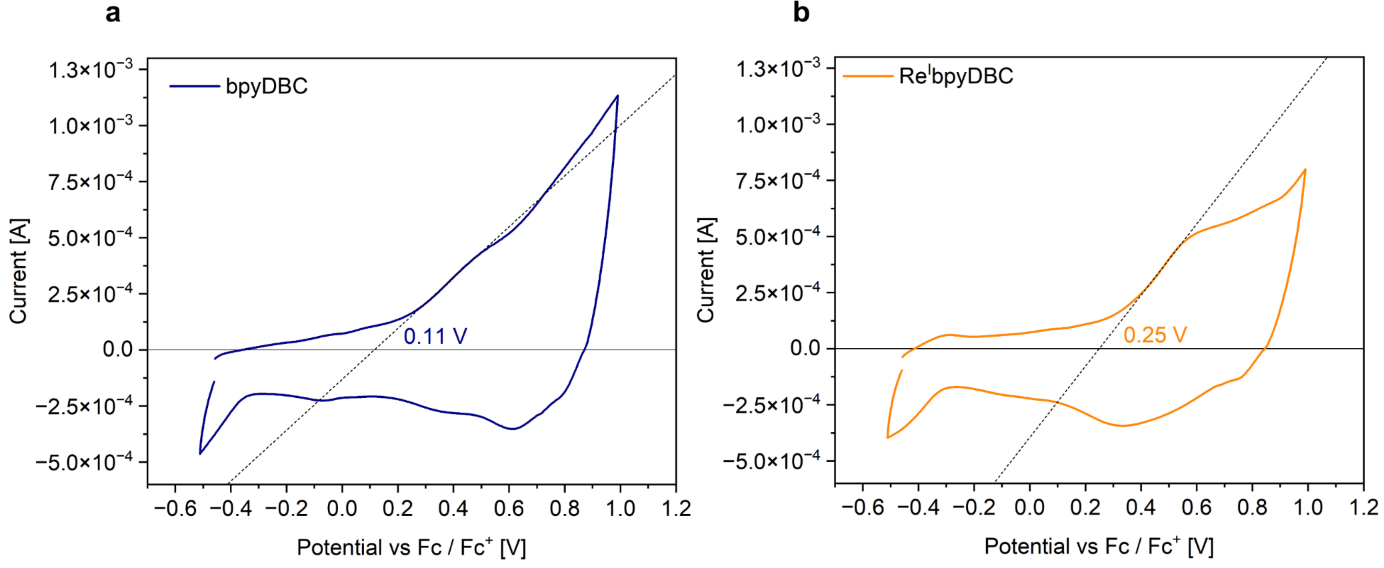


Figure S12. CV curves for both COFs measured against the ferrocene redox couple. The onset of the first oxidation peak reflects the conduction band energy of the material.

## CO_2_ Sorption Experiment


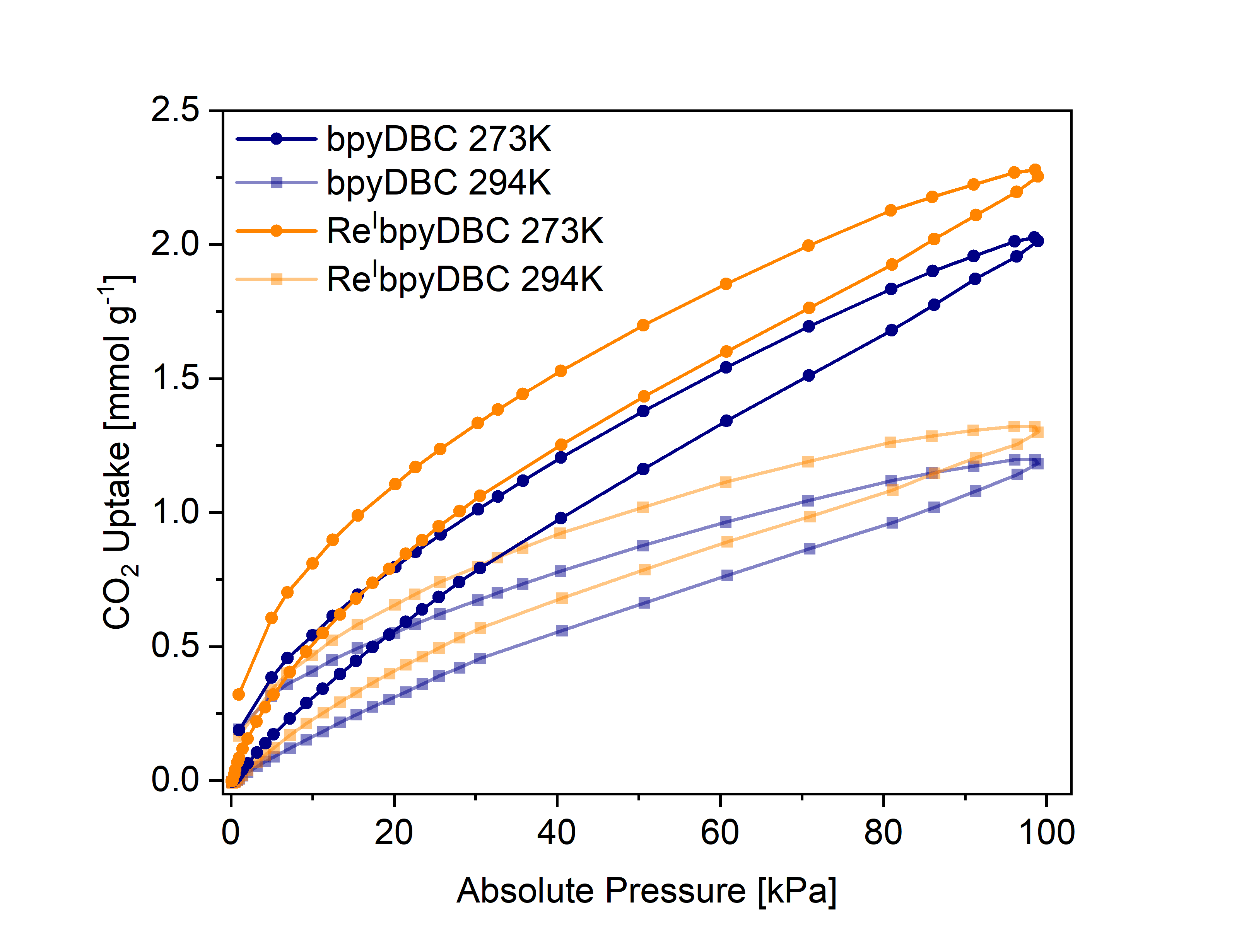


Figure S13. CO_2_ adsoption and desorption of both bpyDBC and Re^I^bpyDBC COF at 273 K and 294 K.

# S5. Photocatalytic experiments

## Reaction scheme of sacrificial donor

Scheme S3. Oxidation process of the sacrificial donor BIH in the photochemical CO_2_ reduction. PS stands for photosystem, in this work the Re^I^bpyDBC COF.

## Comparison of photocatalytic performance

Table S2: COF-based hybrid Re^I^ complexes applied in the photoreduction of CO_2_ to CO.

| **COF** | **CO production rate [µmol g^-1^h^-1^]** | **Sacrificial agent/ solvent** | **Conc. [mg mL^-1^]** | **Amount of Re^I^ active sites [µmol]*** | **TON_CO_** | **Light source** | **Ref.** |
| --- | --- | --- | --- | --- | --- | --- | --- |
| Re^I^bpyDBC | 1160 | BIH/CH_3_CN | 0.3 | 0.93 | 35 (24h, after two additional purges with CO_2_) | 300 W (Xe) >370 nm | [This work] |
| Re-Bpy-sp^2^-c-COF | 1040  1400^a^ | TEOA/ CH_3_CN | 0.2 | 0.97 | 18.7 (17.5 h) | 300 W (Xe) >420 nm | ^[6]^ |
| Re-COF | 750 | TEOA/ CH_3_CN | 0.3 | 0.27 | 48 | 225 W (Xe) >420 nm | ^[7]^ |
| Re-TpBpy-COF | 282 | TEOA/ CH_3_CN-H_2_O | 1.3 | 21.3 | - | 200 W (Xe) >390 nm | ^[8]^ |
| Re^I^bpyDBC | 180 | BIH/CH_3_CN | 0.3 | 0.93 | 7.0 (72 h) | 300 W (Xe) >420 nm | [This work] |

^a^ In the presence of a photosensitizer

* The Re^I^ content was calculated based on the wt% given by ICP OES. For example, in this work, 1 mg of COF used in the photocatalytic experiments contains 16.91 wt% Re, or 169.1 µmol. Divided by the molar mass of Re (182.21 g mol^-1^), a Re content of 0.93 µmol is determined.

Table S3. Comparison of the duration of the photocatalytic experiments for several COF-based CO_2_ photocatalysts.

| **COF** | **Duration of photocatalytic experiment [h]** | **Ref.** |
| --- | --- | --- |
| Re^I^bpyDBC (> 400 nm) | 72 | [This work] |
| PdIn@N_3_-COF | 24 | ^[9]^ |
| Re-COF | 22.5 | ^[7]^ |
| COFdpy-Co | 18 | ^[10]^ |
| Re-Bpy-sp^2^c-COF | 17.5 | ^[6]^ |
| Re-TpBpy-COF | 12 | ^[8]^ |

## ^13^CO_2_ isotope labelling experiments

- **λ > 370 nm**


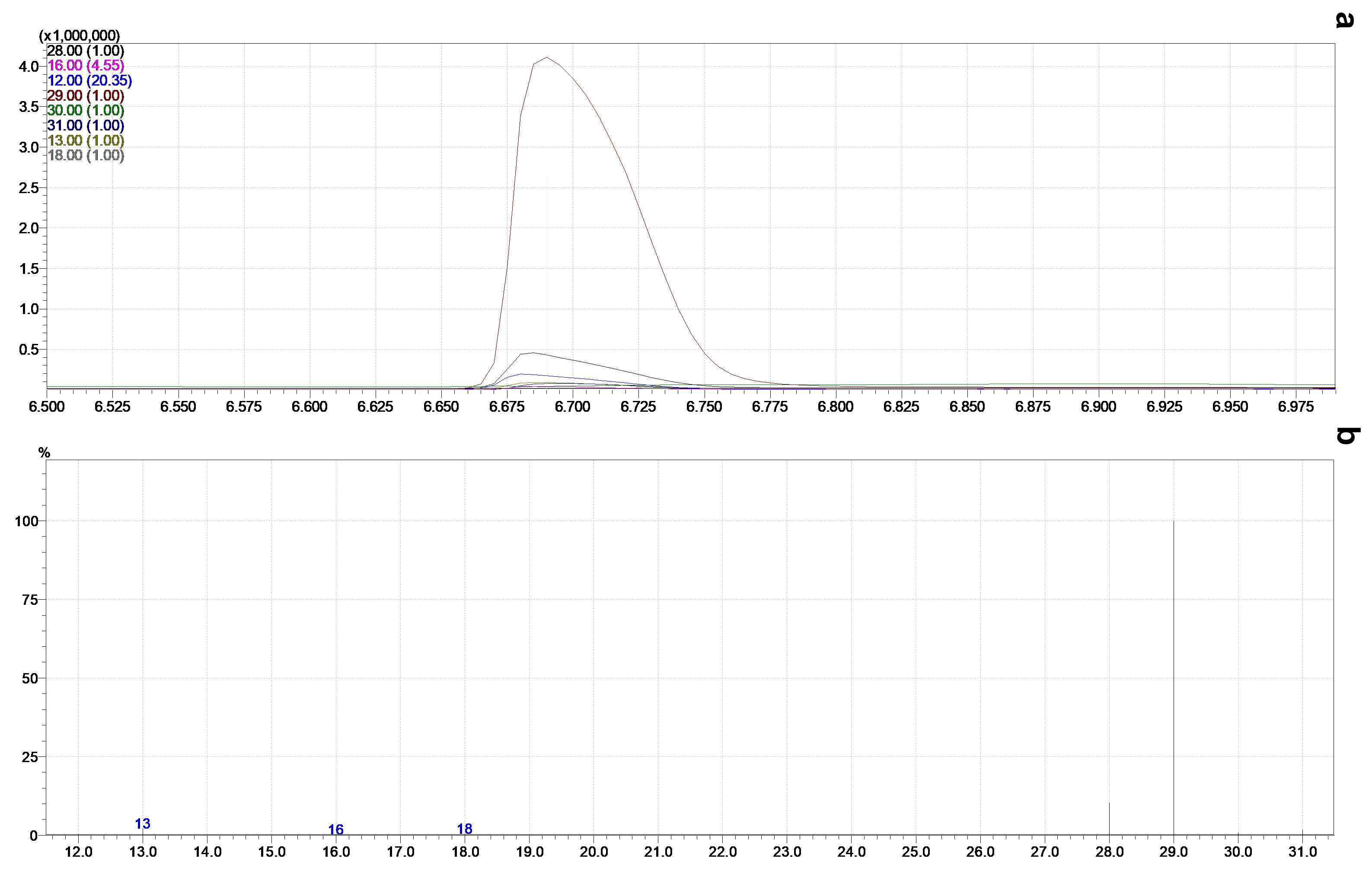


Figure S14. GC-MS analysis of photocatalytic experiments under an illumination of λ > 370 nm using isotopically labelled ^13^CO_2_. (a) Gas chromatography (GC) data showing the retention time of the detected CO and (b) mass spectrometry (MS) confirming the isotopic signature of ^13^CO (M = 29 g mol^-1^), indicating its origin from ^13^CO_2_.

- **λ > 400 nm**


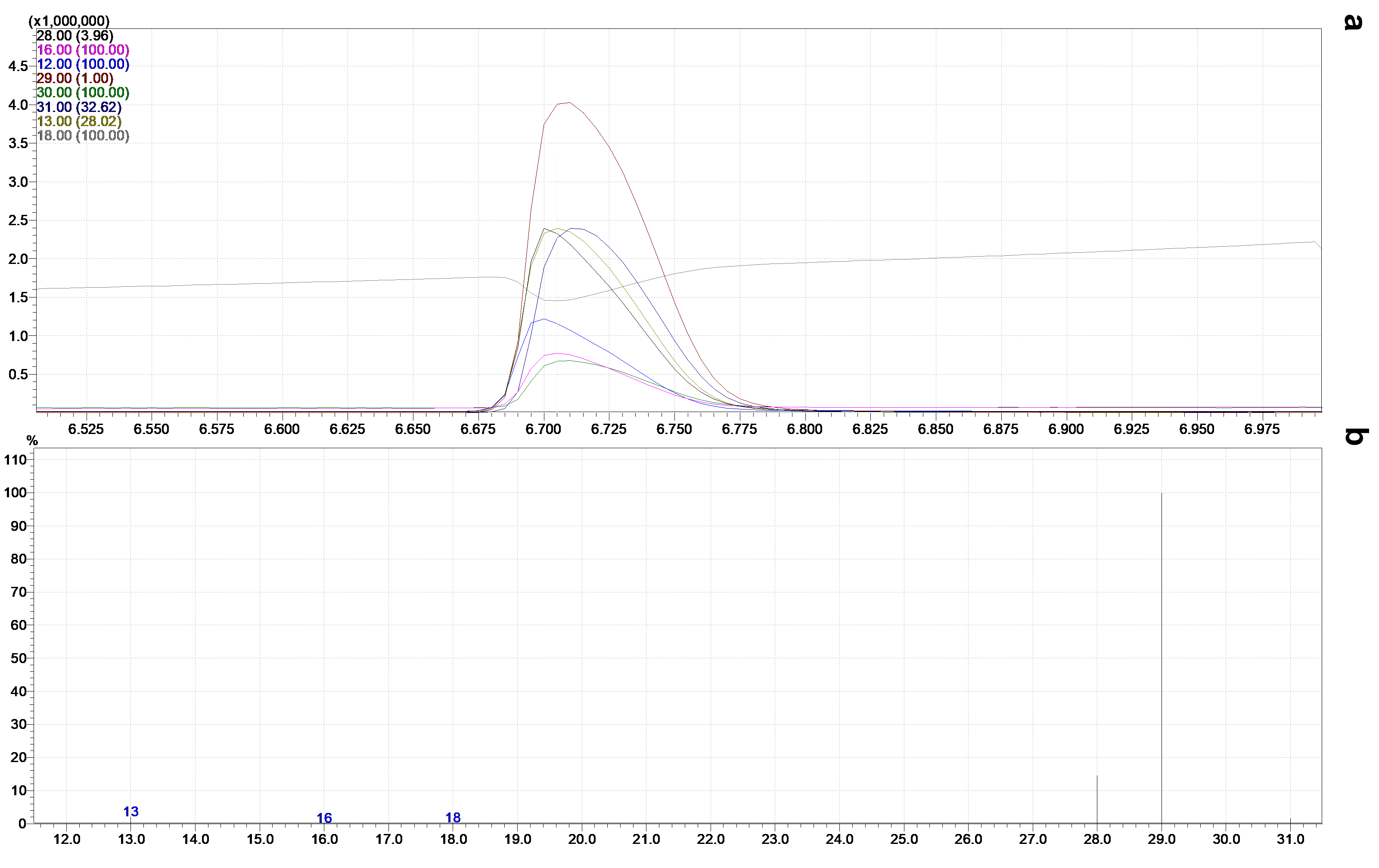


Figure S15. GC-MS analysis of photocatalytic experiments under an illumination of λ > 400 nm using isotopically labelled ^13^CO_2_. (a) Gas chromatography (GC) data showing the retention time of the detected CO and (b) mass spectrometry (MS) confirming the isotopic signature of ^13^CO (M = 29 g mol^-1^), indicating its origin from ^13^CO_2_.

## Recycling studies


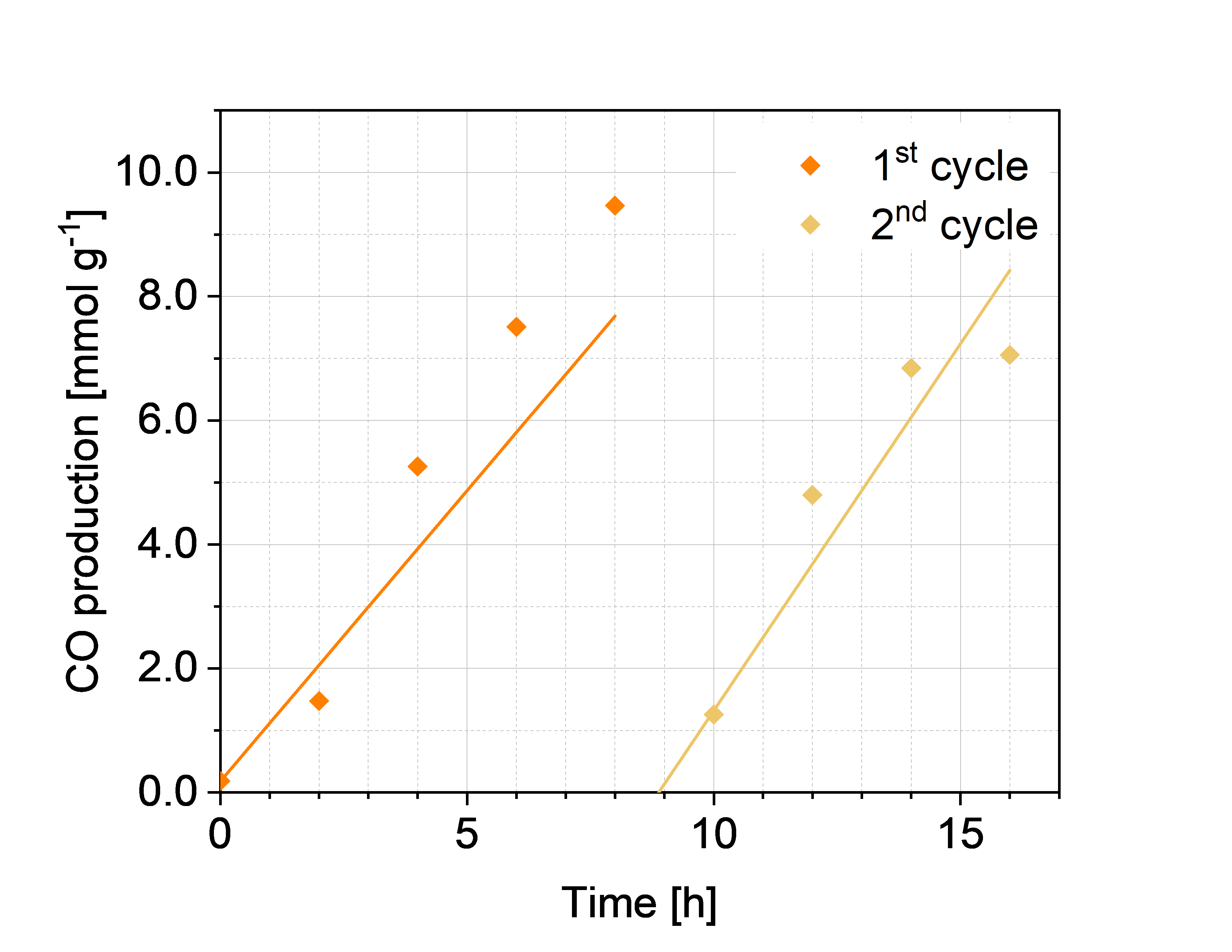


Figure S16: Recycling studies under illumination with λ > 370 nm. Straight lines indicate the linear fitting to extract the CO production rates of 0.94 mmol g^-1^ h^-1^ and 1.18 mmol g^-1^ h^-1^ for the first and second cycle, respectively. The observed plateau in CO production at the end of the second cycle can be attributed to the depletion of BIH, the sacrificial donor.

S6. References

[1] S. Trasatti, *Pure and Applied Chemistry* **1986**, *58*, 955.

[2] P. R. Roberge, *Handbook of corrosion engineering*, McGraw-Hill, New York, **2013**.

[3] N. Keller, T. Sick, N. N. Bach, A. Koszalkowski, J. M. Rotter, D. D. Medina, T. Bein, *Nanoscale* **2019**, *11*, 23338.

[4] X.-Q. Zhu, M.-T. Zhang, A. Yu, C.-H. Wang, J.-P. Cheng, *Journal of the American Chemical Society* **2008**, *130*, 2501.

[5] B. H. Toby, R. B. von Dreele, *J Appl Crystallogr* **2013**, *46*, 544.

[6] Z. Fu, X. Wang, A. M. Gardner, X. Wang, S. Y. Chong, G. Neri, A. J. Cowan, L. Liu, X. Li, A. Vogel et al., *Chemical science* **2020**, *11*, 543.

[7] S. Yang, W. Hu, X. Zhang, P. He, B. Pattengale, C. Liu, M. Cendejas, I. Hermans, X. Zhang, J. Zhang et al., *Journal of the American Chemical Society* **2018**, *140*, 14614.

[8] S.-Y. Li, S. Meng, X. Zou, M. El-Roz, I. Telegeev, O. Thili, T. X. Liu, G. Zhu, *Microporous and Mesoporous Materials* **2019**, *285*, 195.

[9] Y. Huang, P. Du, W.-X. Shi, Y. Wang, S. Yao, Z.-M. Zhang, T.-B. Lu, X. Lu, *Applied Catalysis B: Environmental* **2021**, *288*, 120001.

[10] Y. Xiang, W. Dong, P. Wang, S. Wang, X. Ding, F. Ichihara, Z. Wang, Y. Wada, S. Jin, Y. Weng et al., *Applied Catalysis B: Environmental* **2020**, *274*, 119096.
